# Marine Collagen Hydrolysates Promote Collagen Synthesis, Viability and Proliferation While Downregulating the Synthesis of Pro-Catabolic Markers in Human Articular Chondrocytes

**DOI:** 10.3390/ijms22073693

**Published:** 2021-04-01

**Authors:** Bastien Bourdon, Frédéric Cassé, Nicolas Gruchy, Pierre Cambier, Sylvain Leclercq, Sarah Oddoux, Antoine Noël, Jérôme E. Lafont, Romain Contentin, Philippe Galéra

**Affiliations:** 1 BIOTARGEN, UNICAEN, Normandie University, 14000 Caen, France; bastien.bourdon@unicaen.fr (B.B.); frederic.casse@unicaen.fr (F.C.); nicolas.gruchy@unicaen.fr (N.G.); 21507655@etu.unicaen.fr (P.C.); contentinr@email.chop.edu (R.C.); 2 Dielen Laboratory, 50110 Tourlaville, France; sarah-oddoux@dielen.fr (S.O.); antoine-noel@dielen.fr (A.N.); 3 Normandy Center for Genomic and Personalized Medicine, Department of Genetics, Caen University Hospital, 14000 Caen, France; 4 Service de Chirurgie Orthopédique, Clinique Saint-Martin, 14000 Caen, France; sylvainleclercq60@gmail.com; 5 CNRS UMR 5305, Laboratory of Tissue Biology and Therapeutic Engineering, University Claude Bernard Lyon 1, Univ Lyon, 69367 Lyon, France; jerome.lafont@ibcp.fr

**Keywords:** human chondrocytes, matrix-associated autologous chondrocyte implantation (MACI), osteoarthritis, collagen, collagen hydrolysates, interleukin-1, catabolic markers, senescence, in vitro repair

## Abstract

Cartilage is a non-innervated and non-vascularized tissue. It is composed of one main cell type, the chondrocyte, which governs homeostasis within the cartilage tissue, but has low metabolic activity. Articular cartilage undergoes substantial stresses that lead to chondral defects, and inevitably osteoarthritis (OA) due to the low intrinsic repair capacity of cartilage. OA remains an incurable degenerative disease. In this context, several dietary supplements have shown promising results, notably in the relief of OA symptoms. In this study, we investigated the effects of collagen hydrolysates derived from fish skin (Promerim^®^30 and Promerim^®^60) and fish cartilage (Promerim^®^40) on the phenotype and metabolism of human articular chondrocytes (HACs). First, we demonstrated the safety of Promerim^®^ hydrolysates on HACs cultured in monolayers. Then we showed that, Promerim^®^ hydrolysates can increase the HAC viability and proliferation, while decreasing HAC SA-β-galactosidase activity. To evaluate the effect of Promerim^®^ on a more relevant model of culture, HAC were cultured as organoids in the presence of Promerim^®^ hydrolysates with or without IL-1β to mimic an OA environment. In such conditions, Promerim^®^ hydrolysates led to a decrease in the transcript levels of some proteases that play a major role in the development of OA, such as Htra1 and metalloproteinase-1. Promerim^®^ hydrolysates downregulated HtrA1 protein expression. In contrast, the treatment of cartilage organoids with Promerim^®^ hydrolysates increased the neosynthesis of type I collagen (Promerim^®^30, 40 and 60) and type II collagen isoforms (Promerim^®^30 and 40), the latter being the major characteristic component of the cartilage extracellular matrix. Altogether, our results demonstrate that the use of Promerim^®^ hydrolysates hold promise as complementary dietary supplements in combination with the current classical treatments or as a preventive therapy to delay the occurrence of OA in humans.

## 1. Introduction

Articular cartilage (AC) is the tissue that covers the ends of the bones in diarthrodial joints. AC is mainly composed of an abundant extracellular matrix (ECM) synthesized by a small proportion of chondrocytes (1–3% of the tissue volume) [1,2,3]. The AC ECM contains several collagens and proteoglycans that provide the tissue with resistance to mechanical loads and shear forces. The most abundant collagens are type II (IIB and IIA isoforms), IX and XI collagens, whereas the most abundant proteoglycan is aggrecan [4]. AC resides in a hypoxic environment and has a very low intrinsic capacity for self-repair [5,6,7]. Thus, chondral defects inevitably lead to osteoarthritis (OA) [8]. Moreover, OA has a multifactorial etiology which encompasses aging, obesity, traumas, genetic predispositions, hormonal status [9,10,11]. Thus, 300 million people worldwide suffer from OA, including approximately 30.8 million people in the United States and 70 million people in Europe [12,13].

OA is a degenerative disease that leads to AC degradation. The disruption of cartilage homeostasis, which is tightly regulated by anabolic and catabolic factors, is one of the main features of OA [3,14,15]. However, soon after cartilage injury, chondrocytes transiently proliferate and synthesize a fibrous ECM composed of atypical molecules, notably type I collagen [14,15]. The synthesized fibrocartilage does not have the same biomechanical properties as hyaline cartilage. Hence, fibrocartilage is rapidly degraded and the subchondral bone undergoes friction, which causes pain.

Inflammatory factors such as interleukin-1 (IL-1) and tumor necrosis factor-alpha (TNF-alpha) play a major role in the progression of the OA process by enhancing the synthesis of ECM-degrading factors such as collagenases, aggrecanases, etc. [14,16,17]. Inflammation, in addition to disrupting cartilage homeostasis, also affects whole joint tissues (synovium, joint ligaments, synovial membrane, bone, etc.), further contributing to OA symptoms.

To date, there is no long-term treatment to regenerate cartilage tissue. At the onset of OA, the therapeutic strategy aims to reduce the pain, stiffness or inflammation using analgesics, non-steroidal anti-inflammatory drugs (NSAIDs) and steroids such as corticosteroids. Intra-articular injections of hyaluronan (HA), glucocorticoids or platelet rich plasma (PRP) attenuate OA symptoms and increase the quality of life of the patient, but only for a transient period after injection [18,19,20]. In the most advanced stages of OA, prosthetic surgery is the only recommended treatment [21,22].

The emergence of complementary therapeutics, such as dietary supplements, appears to be an interesting solution [23]. Collagen hydrolysates have been proposed as nutraceuticals to limit or even restore the quality of cartilage tissue in patients with OA. Further, many studies have shown that collagen hydrolysates may constitute a promising dietary supplement to treat cartilage pathologies [24,25,26]. In 1982, a study showed that oral ingestion of hydrolyzed collagen enhanced symptom improvement in 75% of patients [27,28]. More recently, Jiang and colleagues [29] showed that daily oral intake of collagen peptides for 6 months reduces pain and increases joint mobility in women with moderate knee OA. The use of collagen hydrolysates as dietary supplements may represent an alternative strategy for the management and prevention of OA.

In the present study, we first evaluated the effects of three collagen hydrolysates derived from fish skin (Promerim^®^30 and Promerim^®^60) and fish cartilage (Promerim^®^40) on the viability of human articular chondrocytes (HACs) grown as monolayers. The proliferation, viability and senescence of HACs were assessed both in normoxia and hypoxia. Then, we grew HAC as organoids, as previously, to investigate the effect of Promerim^®^ on hyaline-like cartilage cultured in vitro [15,30,31]. The cartilage quality of the organoids was assessed through studies of gene and protein expressions of hyaline chondrocyte markers. This study identified some molecular mechanisms that may be responsible for the beneficial effects of hydrolyzed collagens on the metabolism of human chondrocytes.

## 2. Results

### 2.1. Promerim^®^30, 40 and 60 Show No Cytotoxic Effects on HACs, Promote Their Viability, Proliferation and Inhibit Cell Senescence

**Figure 1:**
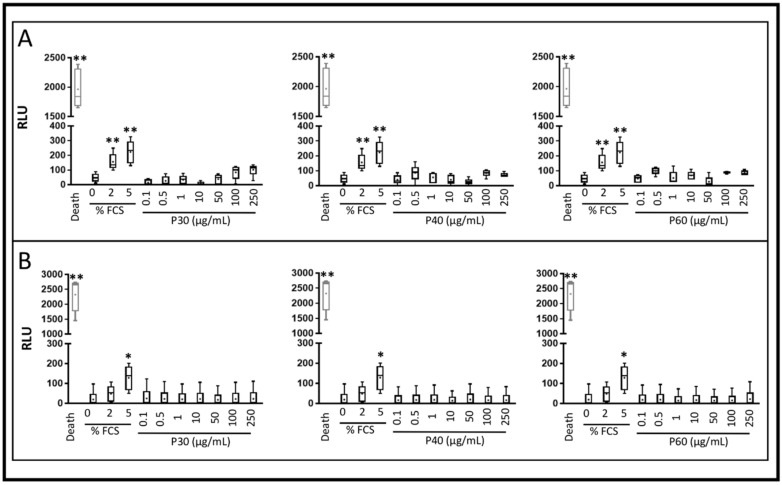
Cytotoxicity of Promerim^®^30, 40 and 60 on human articular chondrocytes. Human articular chondrocytes at P2 and 80% confluency were treated in the absence of FCS, with 0.1, 0.5, 1, 10, 50, 100 or 250 μg/mL of Promerim^®^ hydrolysates for 72 h in hypoxia (**A**) or normoxia (**B**). Controls with 0, 2 and 5% FCS were performed as well as a positive control of 100% cell death (Triton-induced death). Results are summarized in box plots (*n* = 5) showing the mean (cross) and the median (line) of relative light units (RLU) corresponding to the levels of adenylate kinase measured in the media. Statistical analyses were performed using the Mann–Whitney test (* *p* < 0.05; ** *p* < 0.01) compared with the 0% FCS condition.

**Figure 2:**
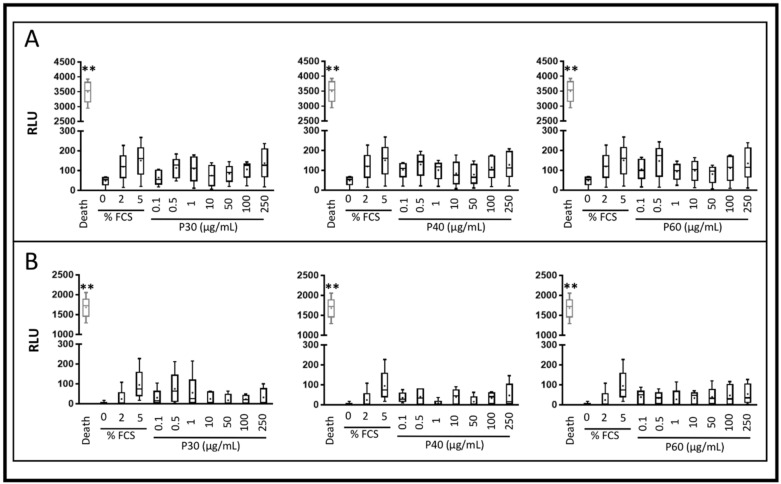
Cytotoxicity of Promerim^®^30, 40 and 60 on human articular chondrocytes. Human articular chondrocytes at P2 and 80% confluency were treated in the presence of 2% FCS, with 0.1, 0.5, 1, 10, 50, 100 or 250 μg/mL Promerim^®^ hydrolysates for 72 h in hypoxia (**A**) or normoxia (**B**). Controls with 0, 2 and 5% FCS were performed as well as a positive control of 100% cell death (Triton-induced death). Results are summarized in box plots (*n* = 5) showing the mean (cross) and the median (line) of relative light units (RLU) corresponding to the levels of adenylate kinase measured in the media. Statistical analyses were performed using the Mann–Whitney test (** *p* < 0.01) compared with the 2% FCS condition.

To evaluate the safety of Promerim^®^ hydrolysates, HACs were cultured in monolayer and at 80% confluency were treated with Promerim^®^30, 40 or 60 at concentrations ranging from 0.1 to 250 µg/mL. Then, the HACs were grown for 72 h, either in a normoxic or hypoxic environment in serum-free media or in the presence of 2% FCS. The hypoxic and the serum-free conditions were used to mimic as much as possible the in vivo chondrocyte microenvironment. We also assessed the safety of Promerim^®^ hydrolysates when HACs were cultured in the presence of 2% FCS, used for optimizing the subsequent organoid culture conditions. The results showed that Promerim^®^ hydrolysates had no cytotoxic effects on HAC regardless of the oxic conditions or serum concentrations used (Figure 1 and Figure 2).

We then studied the effect of Promerim^®^ on the viability and proliferation of HACs in serum-free media or in the presence of 2% FCS.

**Figure 3:**
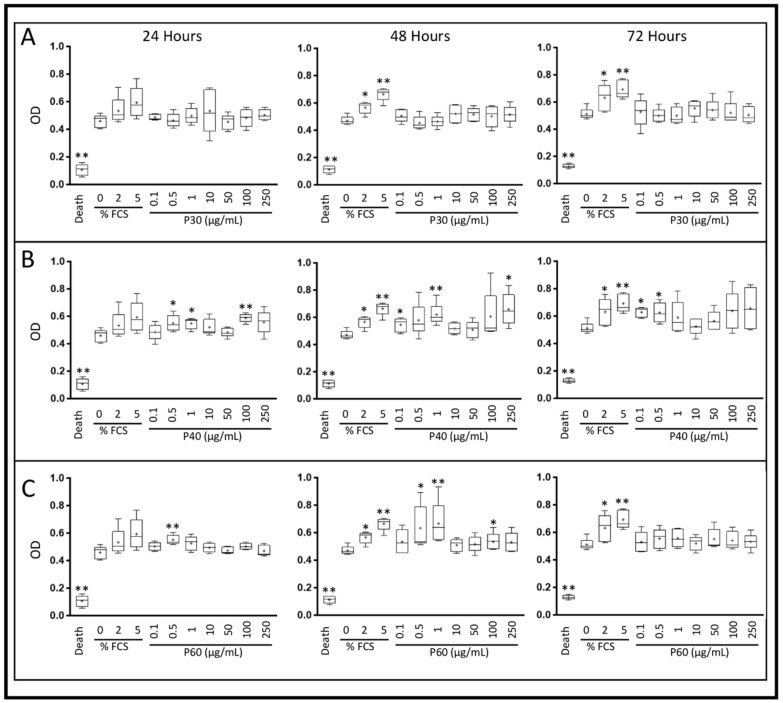
Effect of Promerim^®^ hydrolysates on the viability and proliferation of human articular chondrocytes. Human articular chondrocytes at P2 and 80% confluency were treated in the absence of FCS, with 0.1, 0.5, 1, 10, 50, 100 or 250 μg/mL Promerim^®^30 (**A**), 40 (**B**) and 60 (**C**) for 24, 48 or 72 h in hypoxia. Controls with 0%, 2% and 5% of FCS, and a positive control of 100% cell death (Triton-induced death) were performed. Formazan levels, which are correlated with the viability and proliferation, were measured in the media after 24, 48 or 72 h of treatment. Results are summarized in box plots (*n* = 5) showing the mean (cross) and the median (line) of optical density (OD). Statistical analyses were performed using the Mann–Whitney test (* *p* < 0.05; ** *p* < 0.01) compared with the 0% FCS condition.

In hypoxia and without serum, Promerim^®^30 did not induce any variation in HAC viability and proliferation, compared with the 0% FCS condition (Figure 3A). On the other hand, HAC viability and proliferation increased after 24, 48 and 72 h of treatment with low concentrations of Promerim^®^40 (0.1, 0.5, 1 µg/mL) and after 24 and 48 h of treatment with high concentrations of Promerim^®^40, respectively at 100 and 250 µg/mL (Figure 3B). Promerim^®^60 used at low concentrations (0.5 and 1 µg/mL) increased HAC viability and proliferation after 24 and 48 h of treatment, and only after 48 h of treatment when Promerim^®^60 was used at a higher concentration (100 µg/mL) (Figure 3C).

**Figure 4:**
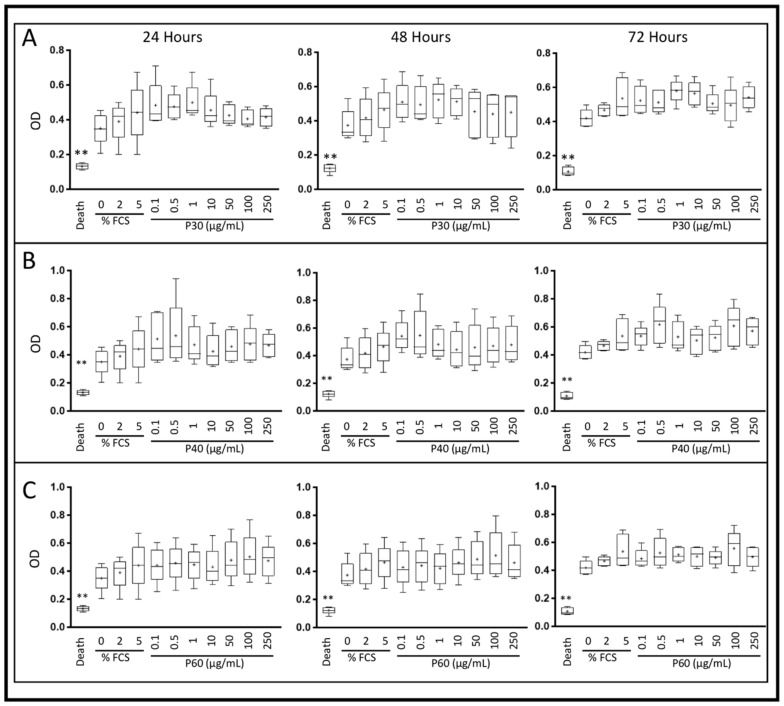
Effect of Promerim^®^ hydrolysates on the viability and proliferation of human articular chondrocytes. Human articular chondrocytes at P2 and 80% confluency were treated in the presence of 2% FCS, with 0.1, 0.5, 1, 10, 50, 100 or 250 μg/mL Promerim^®^30 (**A**), 40 (**B**) or 60 (**C**) for 24, 48 or 72 h in hypoxia. Controls with 0%, 2% and 5% FCS, and a positive control of 100% cell death (Triton-induced death) were performed. Formazan levels, which are correlated with viability and proliferation, were measured in the media after 24, 48 or 72 h of treatment. Results are summarized in box plots (*n* = 5) showing the mean (cross) and the median (line) of optical density (OD). Statistical analyses were performed using the Mann–Whitney test (** *p* < 0.01) compared with the 2% FCS condition.

In the presence of 2% FCS and in hypoxia, Promerim^®^30 and 40 used at concentrations ranging from 0.1 to 10 µg/mL tended to increase HAC viability and proliferation, but there was no statistically significant difference, regardless of the Promerim^®^ hydrolysate used, between the treatment times of for 24, 48 and 72 h (Figure 4A–C).

In normoxia and in serum-free culture conditions, Promerim^®^30 did not induce any variation in HAC viability and proliferation (Figure S1A). In contrast, Promerim^®^40 induced an increase in the viability and proliferation at high concentrations (100 and 250 µg/mL) after 24 h of treatment, at low concentrations (0.1 to 1 µg/mL) after 48 h and at high and low concentrations at 72 h of treatment (Figure S1B). Promerim^®^60 showed an overall trend for increased viability and proliferation (Figure S1C). However, a significant increase was observed at 48 h for concentrations of 0.5 and 250 µg/mL and at 72 h for 0.5 µg/mL. In normoxic conditions and in the presence of 2% FCS, there was no significant increase in the viability and proliferation. On the other hand, an overall trend of increase was observed for Promerim^®^40 and 60 after 24 and 48 h of treatment (Figure S2A–C).

**Figure 5:**
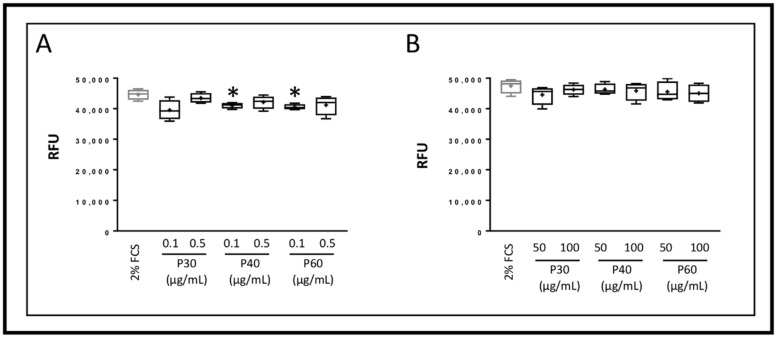
Effect of Promerim^®^30, 40 and 60 on the senescence of human articular chondrocytes. Human articular chondrocytes at P2 and 80% confluency were treated in the presence of 2% FCS, with Promerim^®^30, 40 or 60 (P30, P40 or P60) at 0.1 and 0.5 μg/mL (**A**), 50 and 100 μg/mL (**B**) for 72 h in hypoxia. Controls with 2% FCS were performed (Ctrl) and the levels of β-galactosidase were measured 3 days post-treatment. Results are summarized in box plots (*n* = 4) showing the mean (cross) and the median (line) of relative fluorescence units (RFU) corresponding to the levels of SA-β-galactosidase activity. Statistical analyses were performed using the Mann–Whitney test (* *p* < 0.05) and the control condition was used as the reference.

To investigate the effects of Promerim^®^ on HAC senescence, the cells were cultured in monolayer and treated with Promerim^®^30, 40 or 60 at 0.1, 0.5, 50 or 100 µg/mL for 72 h, in the presence of 2% FCS, either in hypoxia or in normoxia. Then, SA-β-galactosidase activity was assessed to evaluate senescence. In hypoxic conditions, Promerim^®^40 and 60 significantly decreased the activity of SA-β-galactosidase when were used at 0.1 µg/mL (Figure 5A), but this effect was not statistically significant when Promerim^®^40 and 60 were used at higher concentrations (50 and 100 µg/mL) (Figure 5B). Promerim^®^30 used at 0.1 µg/mL with HACs cultured in hypoxia tended to decrease the activity of SA-β-galactosidase, whereas the other concentrations did not affect senescence. In normoxia, Promerim^®^40 used at 0.1 µg/mL significantly decreased SA-β-galactosidase activity (Figure S3A). On the contrary, Promerim^®^30 and 60 did not affect HAC senescence regardless of their concentrations (Figure S3A,B).

In conclusion, our results show that Promerim^®^30, 40 and 60 are not cytotoxic for HACs. Promerim^®^30 and 40 promoted HAC viability and proliferation at high and low concentrations and all Promerim^®^ hydrolysates decreased senescence only at a concentration of 0.1 µg/mL.

### 2.2. Effect of Promerim^®^ on the Expression of mRNAs Typical and Atypical of Articular Chondrocytes

We then evaluated the effects of Promerim^®^30, 40 and 60 on the phenotype of HACs cultured as organoids for 7 days in hypoxia to mimic the in vivo microenvironment of cartilage [15,30,31]. In addition, the HACs were also treated with IL-1β to mimic the pro-inflammatory environment observed during OA. The effects of Promerim^®^ were assessed at high concentrations (50 and 100 µg/mL) and low concentrations (0.1 and 0.5 µg/mL) with or without IL-1β.

**Figure 6:**
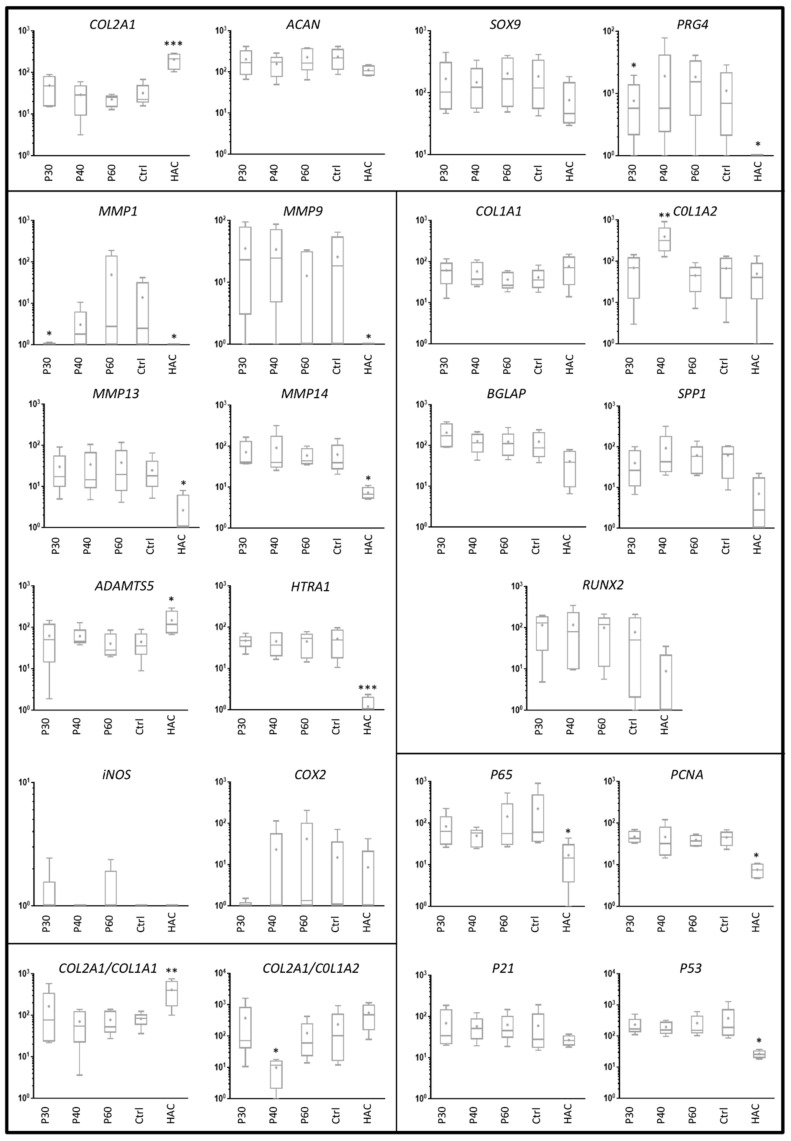
Effect of high concentrations of Promerim^®^30, 40 and 60 on gene expression profile in human articular chondrocytes. Human articular chondrocytes were grown in type I/III collagen sponges at (P2) for 7 days in hypoxia in the absence (Ctrl: control) or presence of 50 µg/mL Promerim^®^30, 40 or 60 (P30, P40, P60). Results are summarized in box plots (*n* = 5) showing the mean (cross) and the median (line) of relative mRNA expression estimated using RT-qPCR and normalized to the *β-ACTIN* and *PPIA* reference genes. The *COL2A1*:*COL1A1* and *COL2A1*:*COL1A2* ratios are given. HAC: mRNA obtained from human articular chondrocytes at P0 were used as controls. Statistical analyses were performed using the two-way ANOVA followed by Dunnett’s multiple comparison post-test (* *p* < 0.05; ** *p* < 0.01; *** *p* < 0.001) compared with the control condition.

**Figure 7:**
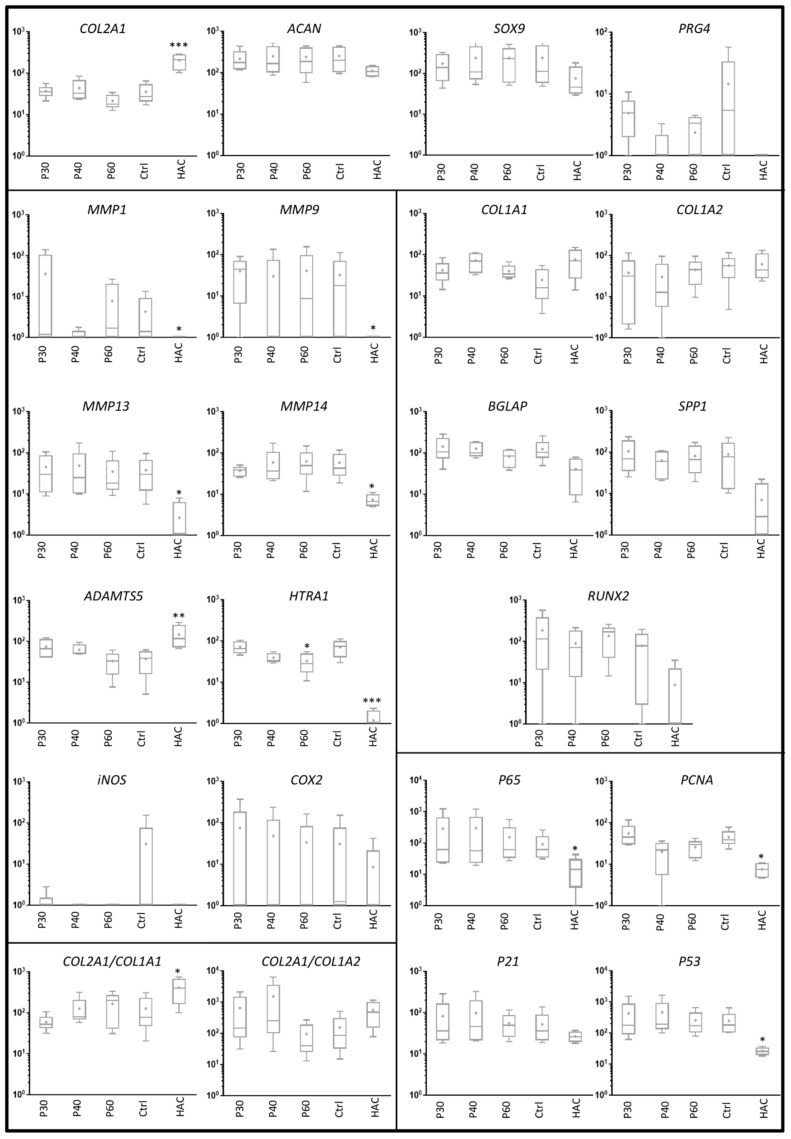
Effect of high concentrations of Promerim^®^30, 40 and 60 on gene expression profile in human articular chondrocytes. Human articular chondrocytes were grown in type I/III collagen sponges at (P2) for 7 days in hypoxia in the absence (Ctrl: control) or presence of 100 µg/mL Promerim^®^30, 40 or 60 (P30, P40, P60). Results are summarized in box plots (*n* = 5) showing the mean (cross) and the median (line) of relative mRNA expression estimated using RT-qPCR and normalized to the *β-ACTIN* and *PPIA* reference genes. The *COL2A1*:*COL1A1* and *COL2A1*:*COL1A2* ratios are given. HAC: mRNA obtained from human articular chondrocytes at P0 were used as controls. Statistical analyses were performed using the two-way ANOVA followed by Dunnett’s multiple comparison post-test (* *p* < 0.05; ** *p* < 0.01; *** *p* < 0.001) compared with the control condition.

When Promerim^®^30 and 60 were used at 50 and 100 µg/mL as well as at 0.1 and 0.5 µg/mL, the steady state transcript levels of chondrocyte phenotypic markers were not statistically different from the control condition (Figure 6 and Figure 7, Figures S4 and S5), except a decrease in the *PRG4* mRNA level with Promerim^®^30 at 50 µg/mL. Among the atypical phenotypic markers, only the *COL1A2* mRNA level increased upon treatment with Promerim^®^40 at 50 µg/mL (Figure 6). Therefore, the *COL2A1*:*COL1A2* ratio decreased (Figure 6). On the contrary, Promerim^®^30 and 40 at 100 µg/mL tended to increase the *COL2A1*:*COL1A2* mRNA ratio (Figure 7).

Because proteases play a major role in OA outcomes, we then investigated their mRNA steady state amounts. Promerim^®^30 (50 µg/mL) decreased *MMP1* mRNA levels and Promerim^®^40 (100 µg/mL) also tended to decrease *MMP1* mRNA amounts (Figure 6 and Figure 7). Promerim^®^60 (100 µg/mL) decreased *HTRA1* mRNA levels (Figure 7). The mRNA levels of the other proteases studied were not modulated in the other Promerim^®^ treatments. The mRNA levels of inflammation-associated molecules (*INOS*, *COX2* and *p65*), proliferative/senescence (*PCNA*, *P53* and *P21*) and hypertrophic markers (*MMP13*, *MMP14*, *BGLAP*, *SPP1* and *RUNX2*) were not statistically modulated either (Figure 6 and Figure 7, Figures S4 and S5).

**Figure 8:**
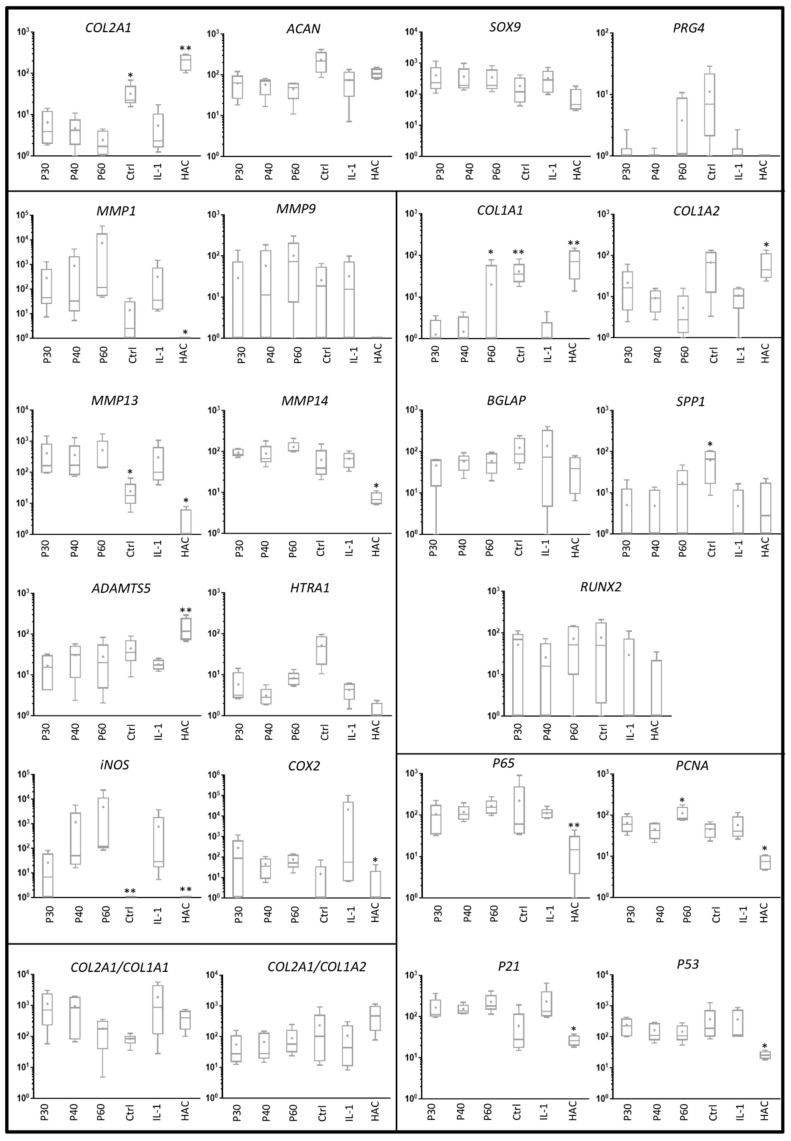
Effect of high concentrations of Promerim^®^30, 40 and 60 on gene expression profile in human articular chondrocytes. Human articular chondrocytes were grown in type I/III collagen sponges at (P2) for 7 days in hypoxia treated with or without IL-1β (10 ng/mL) in the presence of 50 µg/mL Promerim^®^30, 40 or 60 at (P30, P40, P60). Results are summarized in box plots (*n* = 5) showing the mean (cross) and the median (line) of relative mRNA expression estimated using RT-qPCR and normalized to the *β-ACTIN* and *PPIA* reference genes. The *COL2A1*:*COL1A1* and *COL2A1*:*COL1A2* ratios are given. HAC: mRNA obtained from human articular chondrocytes at P0 were used as controls, Ctrl: chondrocytes were grown in type I/III collagen sponges in the absence of IL-1 and Promerim^®^. Statistical analyses were performed using the two-way ANOVA followed by Dunnett’s multiple comparison post-test (* *p* < 0.05; ** *p* < 0.01) compared with the IL-1 condition.

**Figure 9:**
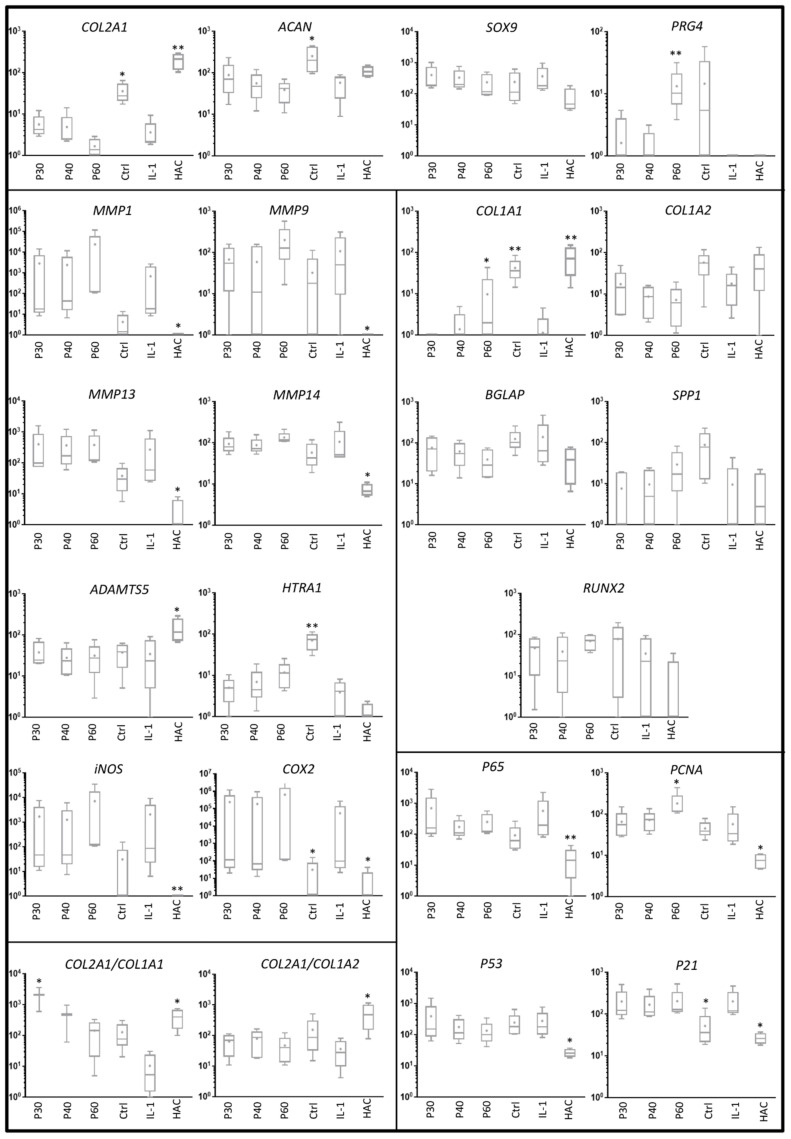
Effect of high concentrations of Promerim^®^30, 40 and 60 on gene expression profile in human articular chondrocytes. Human articular chondrocytes were grown in type I/III collagen sponges at (P2) for 7 days in hypoxia treated with or without IL-1β (10 ng/mL) or the presence of 100 µg/mL Promerim^®^30, 40 or 60 at (P30, P40, P60). Results are summarized in box plots (*n* = 5) showing the mean (cross) and the median (line) of relative mRNA expression estimated using RT-qPCR and normalized to the *β-ACTIN* and *PPIA* reference genes. The *COL2A1*:*COL1A1* and *COL2A1*:*COL1A2* ratios are given. HAC: mRNA obtained from human articular chondrocytes at P0 were used as controls, Ctrl: chondrocytes were grown in type I/III collagen sponges in the absence of IL-1 and Promerim^®^. Statistical analyses were performed using the two-way ANOVA followed by Dunnett’s multiple comparison post-test (* *p* < 0.05; ** *p* < 0.01) compared with the IL-1 condition.

When IL-1β was used to mimic a pro-inflammatory environment, the mRNA levels of typical and atypical HAC markers remained unchanged (Figure 8 and Figure 9), except for *PRG4* and *COL1A1* mRNA levels, which increased when Promerim^®^60 was used at 100 µg/mL and at 50 and 100 µg/mL, respectively (Figure 8 and Figure 9). Promerim^®^30, 40 and 60 at 100 µg/mL tended to increase the levels of the *COL2A1*:*COL1A1* mRNA ratio compared with the IL-1β treated samples. It was particularly statistically significant for Promerim^®^30 (Figure 9). Promerim^®^ hydrolysates, regardless of their concentrations, did not have significant effects on *HTRA1*, *MMP1*, *ADAMTS5*, *INOS*, *COX2* and *p65* mRNA levels (Figure 8 and Figure 9, Figures S6 and S7). Similar results were observed with low or high concentrations of Promerim^®^ on the mRNA levels of hypertrophic markers (*MMP13*, *MMP14*, *BGLAP*, *SPP1* and *RUNX2*) as well as proliferation/senescence-associated molecules (*PCNA*, *P53* and *P21*) (Figure 8 and Figure 9, Figures S6 and S7), except with Promerim^®^60 used at 50 and 100 µg/mL which induced a significant increase in *PCNA* mRNA levels (Figure 8 and Figure 9).

Altogether, our results show that Promerim^®^30, 40 and 60 used at low concentrations (0.1 and 0.5 µg/mL) do not modulate the mRNA levels of chondrocyte phenotype markers, proteases or inflammation and proliferation molecules. Interestingly, higher concentrations of Promerim^®^30 and 60 can induce a significant decrease in protease (i.e., *MMP1* and *HTRA1*) mRNA levels, respectively. Additionally, under pro-inflammatory conditions, Promerim^®^30, 40 and 60 tended to increase the *COL2A1*:*COL1A1* mRNA ratio and Promerim^®^60 increased *PCNA* mRNA amounts.

### 2.3. Effect of Promerim^®^ Hydrolysates on the Expression of Type II and I Collagen Proteins and Htra1

We then evaluated the impact of Promerim^®^ treatments on the newly synthesized ECM in our cartilaginous organoids, by assaying the protein levels of types IIB, II, I and X collagens as well as the serine protease Htra1.

**Figure 10:**
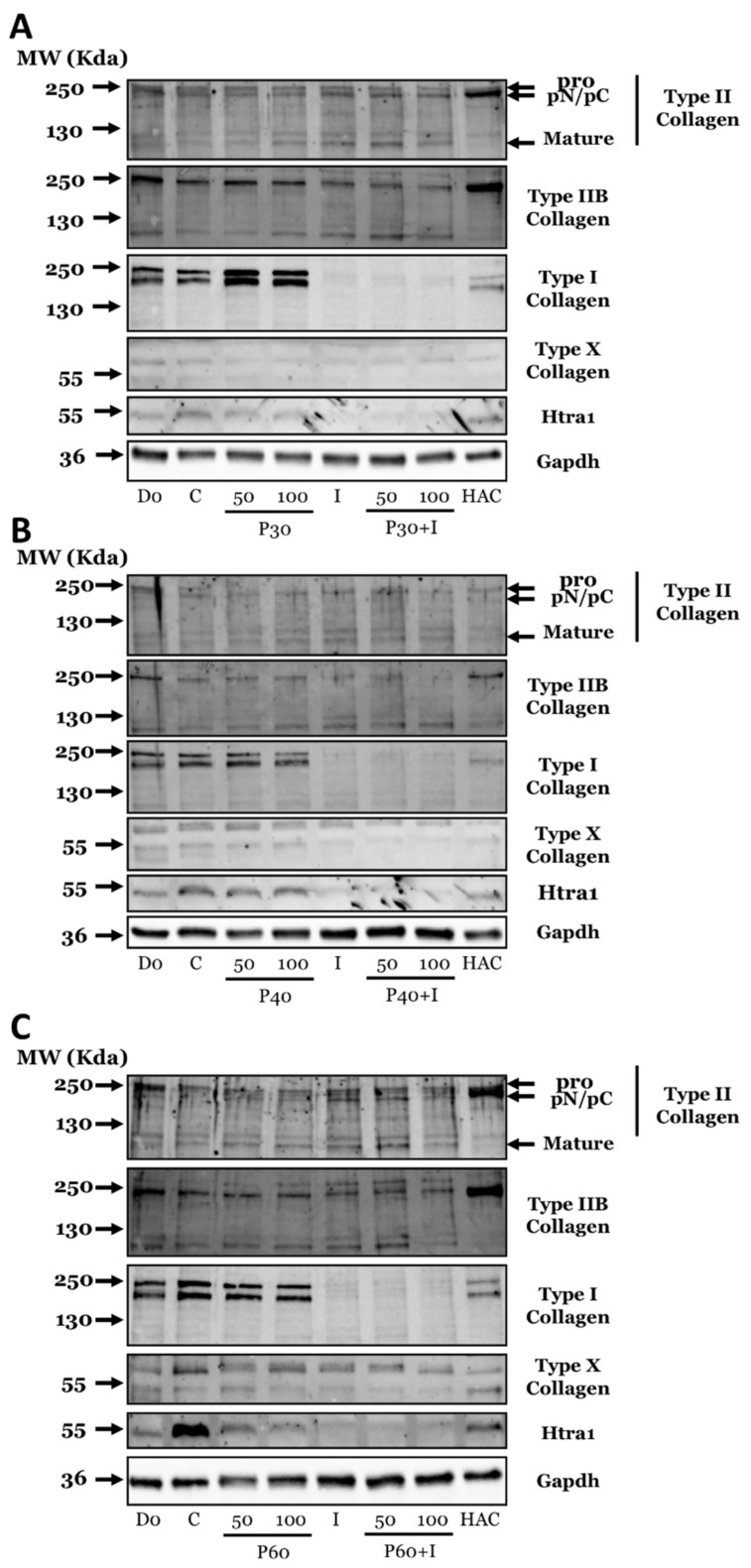
Effect of high concentrations of Promerim^®^ hydrolysates on collagens and HtrA1 protein expression of human articular chondrocytes. Human articular chondrocytes at P2 were inoculated in collagen sponges and treated for 7 days in the absence or presence of IL-1β (10 ng/mL) with Promerim^®^30 (**A**), 40 (**B**) or 60 (**C**) at 50 and 100 µg/mL. The 3D control culture medium was used as control (Ctrl), I (IL-1), P30 (Promerim^®^30), P40 (Promerim^®^40), P60 (Promerim^®^60). Cells seeded in sponges stopped at day 0 (D0) and protein extracts obtained from human articular cartilage (HAC) are also shown. The molecular weights expressed in kDa are shown on the left-hand side of the panels and the target proteins on the right-hand side of the panels. Images show representative immunoblots from different HAC strains (at least *n* = 3). Full membranes are shown in Figure S11 as well as under-exposed and over-exposed blots in Figures S12 and S13, respectively.

First, Promerim^®^30 did not induce a significant modulation in type II collagen synthesis at any concentration, except at 0.1 µg/mL which led to an increase in total type II and IIB collagen (Figure 10A and Figures S8A, S17A and S18A). Additionally, Promerim^®^30 (50 µg/mL) alone increased the protein level of type IIB collagen (Figure 10A and Figure S17A). Promerim^®^40 alone at 0.1 and 0.5 µg/mL slightly increased the type II and IIB collagens expression, and at 0.1 µg/mL in the presence of IL-1, it enhanced both type II collagen isoforms (Figures S8B and S18B). On the contrary, Promerim^®^40 and 60 at 50 and 100 µg/mL did not modulate the protein accumulation of the type II and IIB collagens, as well as Promerim^®^ 60 at low concentrations (Figure 10B,C, Figures S8B,C, S17B,C and S18B,C). Moreover, the treatment with IL-1β led to a slight decrease in type IIB collagen protein amounts, which was not counteracted by Promerims^®^ (Figure 10A–C and Figure S17A–C). When Promerim^®^ hydrolysates were used at high concentrations, Promerim^®^30 (50 and 100 µg/mL) and Promerim^®^ 40 (50 µg/mL) were the only Promerims^®^ able to increase the type I collagen protein amounts (Figure 10A–C and Figure S17A–C), whereas no modulations were observed with Promerim^®^60 (Figure 10C and Figure S17C). At lower concentrations, Promerim^®^30 (0.1 µg/mL) and 40 (0.1 and 0.5 µg/mL), alone or in presence of IL-1β, increased the type I collagen protein amounts (Figures S8A,B and S18A,B). Promerim^®^60 used at low concentrations slightly increased the type I collagen protein amount (Figures S8C and S18C).

In parallel, we investigated the protein levels of the serine protease Htra1, and we observed a decrease when organoids were cultured in the presence of Promerim^®^30 (50 and 100 µg/mL) (Figure 10A and Figure S17A). This observed decrease was weaker with Promerim^®^40 (Figure 10B and Figure S17B) and much more pronounced with Promerim^®^60 (Figure 10C and Figure S17C). In addition, we found no modulation of Htra1 at lower concentrations Promerim^®^30, 40 and 60 (Figures S8A–C, S14A–C, S15A–C, S16A–C and S18A–C). In the presence of IL-1β, which downregulated Htra1 expression, the three Promerim^®^ hydrolysates did not further modulate this effect (Figure 10, Figures S8, S17 and S18).

Finally, we followed type X collagen expression. The 59 kDa form of type X collagen expected in denatured-reduced conditions, expressed in OA cartilage [32] migrated around 55 kDa. The type X collagen protein amounts remained very low whatever the Promerim^®^ hydrolysate and concentration used, meaning that collagen hydrolysates did not modulate this hypertrophic biomarker in these experimental conditions (Figure 10 and Figure S17).

Altogether, our results show that Promerim^®^30, 40 and 60 at high concentrations (50 and 100 µg/mL) downregulate HtrA1 protein expression. Interestingly, Promerim^®^30 (0.1 µg/mL) and 40 (0.1 and 0.5 µg/mL) increased both type II and IIB collagens expression whereas Promerim^®^30 at 50 µg/mL enhanced collagen IIB isoform. Similarly, Promerim^®^40 under pro-inflammatory condition also enhances type II and IIB collagen syntheses at 0.1 µg/mL. Promerim^®^30 at high concentration and Promerim^®^60 at low concentration induced increase of type I collagen expression. In the presence or absence of IL-1 both Promerim^®^30 and 40 at low concentration enhanced type I collagen biosynthesis.

### 2.4. Promerim^®^30 and 40 Promote Proliferation, Whereas Promerim^®^60 Promotes Migration

**Figure 11:**
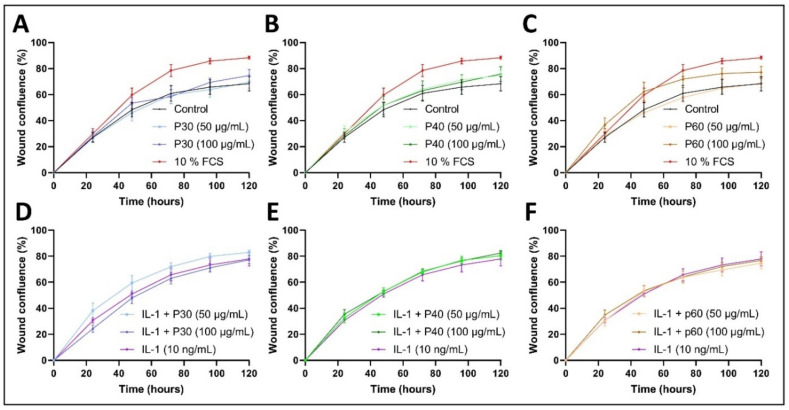
Wound filling analysis following a scratch wound assay. Human articular chondrocytes (HACs) were seeded at P2. At 90% confluency, a scratch wound assay was performed using a WoundMaker^TM^ kit (Essen BioScience) and then the treatments were added. HACs were incubated in the presence of a culture medium containing 2% FCS (Control), and P30, P40, or P60 at 50 and 100 µg/mL without (**A**–**C**) or with IL-1 (**D**–**F**). The wound confluences were calculated using ImageJ software. P30, P40 and P60: Promerim^®^30, Promerim^®^40 and Promerim^®^60 (*n* = 4).

It is of particular interest to favor the proliferation of HACs and promote the migration of HAC and/or progenitor cells to the cartilage lesion in an attempt to delay the outcome of OA. The effects of Promerim^®^30, 40 and 60 on the proliferation/migration of HACs were evaluated using a wound healing test in a 2% FCS environment, as well as in an inflammatory or non-inflammatory environment. In physiological experimental conditions, poor in FCS (2%), Promerim^®^30 (50 and 100 µg/mL) did not increase the speed of wound confluence compared with the control medium (Figure 11A). From 48 to 120 h post-scratch, Promerim^®^40 tended to slightly increase cell confluence in the wound area at concentrations of 50 and 100 µg/mL compared with the control condition, but the wound area was not homogeneously filled in (Figure 11B and Figure S9). Interestingly, when HACs were treated with Promerim^®^60 at 100 µg/mL, wound confluence quickly (from 10 h to 48 h post-scratch) tended to be higher than the control and the 10% FCS medium condition (Figure 11C). Afterwards (from 48 to 120 h post-scratch), the wound confluence reached a plateau and dropped lower than that of the 10% FCS condition, but remained higher than the control condition (2% FCS). At 120 h post-scratch, although the wound area was not confluent, spindle-shaped HACs had homogenously colonized the initial wound (Figure S9). To study the effects of Promerim^®^ in an inflammatory environment, we added IL-1β to the culture medium. Promerim^®^30 at 50 µg/mL increased the cell confluence in the wound area, as a function of time, compared with the IL-1β treatment condition (Figure 11D and Figure S10). On the other hand, Promerim^®^40 did not seem to have any effect on wound healing in a pro-inflammatory environment compared with HACs treated with IL-1β only (Figure 11E and Figure S10). Promerim^®^60 did not increase wound confluence, but at 72 h post-scratch, although the wound area was not confluent, it was homogeneously filled with spindle-shaped HACs, suggesting migration of HACs rather than proliferation (Figure 11F and Figure S10).

Altogether these data suggest that Promerim^®^40 (basal condition) and Promerim^®^30 (under inflammatory conditions) promote proliferation, whereas Promerim^®^60 promotes migration with or without inflammatory conditions, even though migratory kinetics are faster under inflammatory conditions.

## 3. Discussion

Numerous of food supplements are available without prescription in pharmacies and health food stores to treat OA [23,33]. Among the most frequently used active elements, collagen hydrolysates [34], *Boswellia serrata*, *Curcuma longa* [35] and curcumin extract [36,37], have demonstrated clinically significant short-term reduction in pain [38]. Other nutraceuticals, such as glucosamine and chondroitin, avocado and unsaponifiable soy [39] or undenatured type II collagen [40], have been shown to significantly relieve discomfort associated with OA [41,42,43], but their mechanisms and long-term effects on cartilage regeneration and inflammation are still uncertain [38]. The efficacity of collagen hydrolysates has been studied in human clinical trials. In one trial with women suffering from moderate knee OA, daily oral intake of collagen peptides for 6 months increased the mobility of the affected joint and reduced pain [29]. Other clinical trials also showed improvement in joint functionality and discomfort with a daily intake of collagen hydrolysate-based nutritional supplements for periods ranging from 3 months to one year [24,44,45]. Thus, collagen hydrolysates seem to be able to reduce OA symptoms or delay its outcomes through mechanisms that mainly involve the decrease of cartilage degradation or inflammation [46].

In the present study, we evaluated the effects of three collagen hydrolysates derived from fish skin (Promerim^®^30 and Promerim^®^60) and fish cartilage (Promerim^®^40) on the viability, proliferation, senescence and the phenotype of HACs grown as cartilaginous organoids.

We firstly demonstrated that Promerim^®^ hydrolysates have no cytotoxic effects on HACs. We then investigated HAC viability and proliferation since the downregulation of both these two mechanisms, mitochondrial dysfunction, and damage are classic hallmarks of aging [47]. In OA, mitochondrial dysfunction may alter AC metabolism, function and viability, and hence contribute to cartilage degeneration [7,48,49,50]. These dysfunctions have an impact on chondrocyte anabolism and growth responses. In addition, they exacerbate oxidative stress and apoptosis of chondrocytes, as well as the inflammatory responses induced by IL-1β and TNFα on ECM catabolism [7,47,48,49]. Here, we demonstrated that Promerim^®^ hydrolysates can increase the viability and proliferation, which make them useful to improve chondrocyte metabolism.

During OA pathogenesis, the senescence of chondrocytes is a key element that plays a significant role in the pathological process and precedes the stages prior to subchondral bone exposure. Numerous factors such as obesity, age, mechanical failure, oxidative stress as well as, at the cellular level, dedifferentiation or apoptotic processes can induce senescence [51,52,53,54,55]. Studies have reported that disease severity of patients with knee OA correlates with senescence-associated β-galactosidase activity [56], and the suppression of senescent chondrocytes delays the progression of induced OA in a mouse model [57]. Thus, by inducing a decrease in HAC senescence, Promerim^®^ may help delay the progression of OA.

OA is a progressive disease characterized by sequential steps, which start after an initiating event that induces an increase in intra-articular inflammation followed by the induction of several enzymes, such as aggrecanases, matrix metalloproteinases and serine proteases such as Htra1. Htra1 is upregulated in the synovial fluid and AC in patients with OA and rheumatoid arthritis and plays a critical role in disease development [58]. For example, it contributes, directly or indirectly via the upregulation of metalloproteinases, to the degradation of ECM components, and inhibits anabolic signaling by antagonizing the receptors of the chondrogenic TGF-β family [59]. Here, we demonstrated that when a pro-OA environment is mimicked with IL-1ß treatment, Htra1 is systematically downregulated in HACs grown as 3D organoids. Interestingly, Promerim^®^ (Promerim^®^30 and 60) induced a decrease in *HTRA1* and *MMP1* transcript levels, which is of particular interest to limit cartilage degradation that occurs in OA and to favor anabolism, notably by potentially restoring TGF-β signaling.

In our study, the mRNA levels of *COL2A1*, *ACAN* and *COL1A1* were decreased in the presence of IL-1, whereas those of some inflammation mediators, *iNOS* and *COX2*, and of some proteases (*MMP1* and *MMP13*) were enhanced. By contrast, the level of *ADAMTS5*, known to degrade aggrecan during OA, remained unchanged. Our results suggest that the human articular chondrocytes we used remained sensitive to IL-1, at least in part, even though IL-1 receptor desensitization could have occurred when chondrocytes were exposed for a long period to pro-inflammatory signals (human articular chondrocytes used in our study were isolated from OA old patients). It is highly probable that IL-1 effects on HtrA1 expression may vary according to the evolution of the OA process. One can hypothesize that during the early steps of OA, IL-1 enhances HtrA1 expression whereas at the latest stages of this pathology, the cytokine is responsible of the downregulation of this protease, through the involvement of IL-1 receptors desensitization or implication of receptors displaying high or low affinity towards the ligand, responsible for such an ambivalent role of IL-1. Therefore, our results highlight the complexity of the pro-inflammatory microenvironment associated to the numerous cytokines involved in OA pathogenesis. Further studies are needed to fully elucidate the mechanisms by which HtrA1 is upregulated during OA, whereas IL-1 downregulates its expression.

Several cell-based therapies using chondrocytes or mesenchymal stem cells (MSCs) have been developed to reconstitute cartilage of hyaline quality [15,60]. These techniques initially based on the autologous chondrocyte implantation (ACI) procedure developed by Brittberg et al. [60] have been improved by several modifications (hypoxic conditions, culture in 3D biomaterials, use of chondrogenic factors, using different cell sources including MSCs, etc.). However, the quality of the in vitro synthesized cartilage organoid is not strictly identical to healthy hyaline cartilage in vivo. We have previously shown that treatment with *Pecten maximus* extracts on monolayer cultures of chondrocytes or cartilaginous organoids promotes chondrocyte redifferentiation and phenotype maintenance [61]. In this context, Promerim^®^ is a good treatment for promoting hyaline-like ECM production and improving differentiation, as recently demonstrated for equine articular chondrocytes (eACs) [62]. In the present study, we demonstrated that the Promerim^®^ 30 and 40 hydrolysates have the potential to increase collagen protein accumulation (both type II and IIB isoforms as well as type I collagen), even if the conditions to favor these effects differ according to the type of Promerim^®^. Promerim^®^30 and 40 at low concentration increased the collagen protein amounts when organoids were cultured in the presence of IL-1ß. This increase in collagen accumulation can be attributed to the decrease in the expression and activity of several proteases, notably Htra1 with its direct and indirect roles on ECM degradation/synthesis, and for transcriptional upregulation, at least for increases in type I and II collagen protein amounts.

Thus, Promerim^®^30 may be useful during the ACI procedure to increase ECM synthesis during the differentiation/redifferentiation steps, while inhibiting their senescence, and may be useful in vivo for favoring cartilage anabolism at the onset of OA. Promerim^®^40 and 60 may also be useful to delay OA and/or inhibit cartilage degradation when the inflammatory microenvironment occurs, if these results are, of course, confirmed in vivo. The efficacity of Promerim^®^40 and 60 may also be enhanced by their ability to inhibit chondrocyte senescence.

We previously investigated in vitro cell migration and repair using molecules of marine origin with positive effects [62,63]. Therefore, we studied whether Promerim^®^ hydrolysates can promote healing of chondral lesions mimicked in vitro. Scratch wound assays were performed on HACs in a healthy microenvironment or in pro-inflammatory conditions by incubating the HACs in the presence of IL-1β. Promerim^®^40 and 60 led to an increase in cell confluence in the wound area in the absence of IL-1β. This increase was consistent with the enhancement of the viability and proliferation observed in XTT tests. In an inflammatory microenvironment, Promerim^®^30 increased cell confluence in the wound area. Promerim^®^40 in the basal conditions and Promerim^®^30 under inflammatory context seemed to promote proliferation rather than migration, because the wound area was initiated at the edges of the wound, the wound area never became a homogenous layer, and wound confluence did not reach a plateau at the end of the experiments. In contrast, although an increase in PCNA mRNA was observed, Promerim^®^60 seemed to promote migration rather than proliferation, because the wound area was homogenously filled in with spindle-shaped cells, and wound confluence proceeded quickly and reached a plateau before confluence was complete. Therefore, Promerim^®^ hydrolysates may enhance proliferation and migration of chondrocytes in in vivo chondral lesions.

Many nutraceuticals have an antioxidant effect or promote the synthesis of prostaglandin E2 (PGE2) and LT4 pro-inflammatory factors [64,65,66,67]. We have demonstrated that shell extracts of marine origin increase MMP-1 activity in HACs as well as the catabolism of human dermal fibroblasts [61,68]. However, such effects may be different for Promerim^®^ hydrolysates due to their qualitative nature, because shell extracts contain high concentrations of marine minerals. Protein fragments released during enzymatic digestion, as in Promerim^®^, can generate bioactive peptides assimilated to matrikines [69]. Indeed, matrikines are currently of major interest in rheumatology and particularly in OA for chondral defect repair [70].

We showed in this study that Promerim^®^ hydrolysates have no cytotoxic effect on HACs, but promote HAC viability and proliferation, and even decrease HAC senescence. Interestingly, Promerim^®^ hydrolysates in cartilaginous organoids increased collagen production and also favored ECM production, which subsequently improved the quality of cartilaginous organoids. Furthermore, Promerim^®^30 and Promerim^®^60 downregulated several proteases and may have beneficial effects by delaying ECM degradation and inflammation. Overall, Promerim^®^ hydrolysates hold promise as nutraceuticals with promising effects to prevent or delay OA pathogenesis.

The equine sector also suffers from large financial losses due to OA and tendinopathies in racehorses. Indeed, the OA degenerative pathology is the source of lameness in horses and causes the early termination of their racing career. Human and horse cartilage share many similarities and the nature of their chondral lesions are also similar. We have carried out similar studies in an equine AC model [62] and have shown that Promerim^®^30, 40 and 60 are able to induce an increase of eAC viability and proliferation. In the present study on HACs, Promerim^®^30 did not increase HAC viability and proliferation. Additionally, whereas only Promerim^®^30 decreased eAC senescence, Promerim^®^40 and 60 were also able to decrease the senescence of HACs. In our study on eACs, Promerim^®^ hydrolysates decreased the mRNA levels of a larger panel of proteases, notably Mmp3 and Adamts5. Furthermore, when we treated eAC organoids with Promerim^®^, the increase in collagen protein amounts was sharper. In particular, the pro-inflammatory microenvironment systematically enhanced the effect of Promerim^®^. Thus, Promerim^®^ seems to be less effective on HACs than on eACs in the treatment of OA. We do not attribute the discrepancy of Promerim^®^ affects to species differences but rather to the nature of the cells, and more especially the age of the cell sources used for the experiments. In our previous study on eACs we isolated eACs from young healthy horses (between 4 and 10 years old), whereas the HACs used in the present study come from older patients that had undergone hip replacement surgery. Thus, eACs were isolated from hyaline cartilage, whereas HACs were isolated from OA cartilage, thus exposed for a long period to very high levels of pro-inflammatory cytokines such as IL-1β and TNF-α. These cytokines likely hyperactivate dedifferentiation and catabolic behaviors, preventing any further positive chondrogenic effect. Therefore, although Promerim^®^ may be useful at later stages of OA in some instances, these hydrolysates are likely more beneficial at the onset of OA, before the first symptoms occur, as a preventive treatment, or eventually as a companion treatment to current surgical techniques.

## 4. Materials and Methods

### 4.1. Collagen Hydrolysates

Promerim^®^ marine peptides are produced by enzymatic hydrolysis characterized by the control of hydrolysis time, acidity (pH), and temperature. No chemical process of hydrolysis was used. Promerim^®^ correspond to hydrolysates of fish skin and cartilage proteins composed of collagen peptides. These are oligopeptides, that is, small peptides characterized by low molecular weights below 1500 Dalton. They are composed of 2, to a maximum of 15 amino acids. The minimum amount of hydroxyproline is 5 g/100 g and the two major amino acids are proline (9 g/100 g) and glycine (21 g/100 g). Promerims^®^ are produced by the Dielen Laboratory (Tourlaville, France).

### 4.2. Human Articular Chondrocyte Culture

HACs were isolated from osteoarthritic femoral head cartilage obtained from total femoral arthroplasty (40 different patients). The study was performed in full accordance with the local ethics committee guidelines and all the cartilage samples were collected after written and informed consent of the donors according to French legislation. All the experimental protocols were approved by the French Ministry of Higher Education and Research (Ethics Committee for Research on Human Samples CODECOH: DC 2014–2325, 20 October 2015).

The cartilage was separated from the subchondral bone and cut into small pieces using a sterile razor blade. Cartilage pieces were washed twice with preheated phosphate buffer saline (PBS) at 0.1 M, and digested for 45 min at 37 °C with 20 mg of protease from Streptomyces griseus type XIV (Merck Millipore, Billerica, MA, USA) per gram of cartilage tissue. The cartilage was rinsed twice with PBS, and then incubated overnight at 37 °C with 20 mg of type I collagenase from Clostridium histolyticum (Thermo Fisher Scientific, Waltham, MA, USA) per gram of cartilage tissue. The protease solutions were prepared at 2 mg/mL in high-glucose (4.5 g/L) Dulbecco’s modified Eagle’s medium (DMEM, Eurobio Scientific, Courtaboeuf, France) supplemented with 100 IU/mL penicillin, 100 µg/mL streptomycin, and 0.25 µg/mL amphotericin B (Eurobio Scientific, Courtaboeuf, France). The next day, the cell suspension was filtered through a 70 μm cell strainer, centrifuged at 300× *g* for 10 min and plated in culture flasks at a density of 20,000 cells/cm^2^. Cells were cultured in chondrocyte growth medium containing DMEM supplemented with 10% fetal calf serum (FCS, Eurobio Scientific, Courtaboeuf, France), 100 µg/mL streptomycin, 100 IU/mL penicillin and 0.25 µg/mL amphotericin B in humidified atmosphere at 37 °C, 5% CO_2_. The medium was changed twice a week until reaching 95% confluency. For the first cell passage (P1), chondrocytes were collected by trypsinization with 0.05% trypsin/1 mM EDTA (Eurobio Scientific, Courtaboeuf, France), counted, centrifuged at 300× *g* for 10 min and plated in culture flasks at a density of 20,000 cells/cm^2^.

### 4.3. 3D Model for Chondrocyte Redifferentiation

Sponges 2 mm thick and 5 mm in diameter composed of native type I collagen (90–95%) and type III collagen (5–10%) from calf skin (Symatèse Biomatériaux, Chaponost, France) were used for redifferentiation studies. Briefly, at 95% confluency, HACs from P1 were harvested by trypsinization with 0.05% trypsin/1 mM EDTA (Eurobio Scientific, Courtaboeuf, France), counted, centrifuged at 300× *g* for 10 min and seeded onto the sponges as previously described [31,32]. The next day, cells were incubated in DMEM supplemented with antibiotics, 50 µg/mL ascorbic acid-2-phosphate and 2% FCS in the presence or absence of IL-1β (10 ng/mL) and/or Promerims^®^. 3D cultures were maintained in hypoxia (3% O_2_) at 37 °C and 5% CO_2_ in a sealed chamber for 7 days, and the media were changed on day 3. At the end of the experiment, sponges were washed twice in preheated PBS and frozen at −80 °C until future analysis.

### 4.4. Chondrocyte Toxicity Assay

HACs were seeded at passage 2 (P2) in 96-well plates at a density of 20,000 cells/cm^2^. When 80% confluency was reached, the cells were treated in the absence or presence of FCS (2%), with 0.1, 0.5, 1, 10, 50, 100 or 250 μg/mL Promerim^®^ hydrolysates for 72 h in hypoxia or normoxia. After 72 h, 80 µL of the supernatant was collected to measure the levels of adenylate kinase (AK) released from damaged cells with a cytotoxicity detection kit (ToxiLight™ bioassay kit, Interchim, Montluçon, France). The maximum chondrocyte death rate was induced in sister wells exposed to Triton X-100 for 10 min. Bioluminescence was measured using a Spark^®^ multimode microplate reader (Tecan^®^) in white flat-bottom 96-well plates.

### 4.5. HAC Viability and Proliferation Measurement

HACs were seeded at P2 in 96-well plates at a density of 20,000 cells/cm^2^. When 80% confluency was reached, the cells were treated in the absence or presence of FCS (2%), with 0.1, 0.5, 1, 10, 50, 100 or 250 μg/mL Promerim^®^ hydrolysates for 24, 48 and 72 h in hypoxia or normoxia. At the end of the experiment, viability and proliferation were determined using the cell proliferation kit II XTT (Roche). Briefly, 50 µL of XTT labelling reagents was added per well and incubation took place for 1.25 h at 37 °C in a cell culture incubator. Absorbance was measured directly in the culture plate using a Spark^®^ multimode microplate reader (Tecan^®^) at 450 nm with a reference wavelength at 600 nm.

### 4.6. Senescence-Associated β-Galactosidase Quantification

HACs were seeded at P2 in 96-well plates at a density of 20,000 cells/cm^2^. When 80% confluency was reached, the cells were treated in the presence of FCS (2%), with 0.1, 0.5, 50 or 100 μg/mL Promerim^®^ hydrolysates for 72 h in hypoxia or normoxia. At the end of the experiment, the β-galactosidase activity was measured using the 96-well cellular senescence assay (SA β-Gal Activity) kit (Cell Biolabs, San Diego, CA, USA) as described by the manufacturer. Briefly, after one wash with cold PBS, cells were lysed in lysis buffer at 4 °C for 5 min and centrifuged at 13,000× *g* for 10 min. Supernatants were transferred to 96-well plates and incubated at 37 °C for 16 h with the assay reagents. The reaction mixture was transferred into a white flat-bottom 96-well plate and with the addition of the stop solution. The fluorescence was measured using a Spark^®^ multimode microplate reader (Tecan^®^) at 465 nm with a wavelength excitation at 360 nm.

### 4.7. RNA Extraction and Quantitative Real-Time PCR

Total RNA was extracted in 250 µl of RNA-Solv^®^ Reagent (Omega Biotek, Norcross, GA, USA) per sponge according to the manufacturer instructions. After removal of the potential genomic DNA using DNase I (Fisher, Illkrich, France) for 10 min followed by inactivation with EDTA for 10 min at 65 °C, total RNA (1 µg) from each sample was reverse-transcribed into cDNA using the iScript™ Reverse Transcription Supermix (Bio-Rad, Hercules, CA, USA). Quantitative real-time PCR was performed on a CFX96 touch Real Time PCR Detection System (Bio-Rad, Hercules, CA, USA) using GoTaq^®^ qPCR Master Mix (Promega, Madison, WI, USA). The 2^-ΔΔCT^ method was used to calculate, with CFX ManagerTM software 3.1 (Bio-Rad, Hercules, CA, USA), the relative gene expression with the *ß-ACTIN* and *PPIA* genes as endogenous reference genes. The sequences of the primers (Eurogentec, Liège, Belgium) used are listed in Supplementary Table S1.

### 4.8. Protein Extractions

Sponges containing chondrocytes were frozen and ground and the subsequent extraction of the total proteins was performed using RIPA-lysis buffer supplemented with a cocktail of protease inhibitors (phenylmethylsulfonyl fluoride, pepstatin A, aprotinin and leupeptin) and the phosphatase inhibitor sodium orthovanadate for 45 min at 4 °C as previously described [62]. Protein concentration was determined according to the Bradford procedure (Bio-Rad, Hercules, CA, USA).

### 4.9. Western-Blot

Protein extracts (12 µg per sample) were resolved on 10% polyacrylamide gels (TGX Stain Free Fast Cast Acrylamide Kit 10%, Bio-Rad, Hercules, CA, USA) and transferred to a polyvinylidene difluoride membrane (Trans-Blot Turbo RTA Midi PVDF Transfer Kit, Bio-Rad, Hercules, CA, USA). Membranes were incubated with 10% non-fat milk powder in Tris-buffered saline with 0.1% Tween (TBST) for 1 h to block unspecific binding sites. Then, membranes were incubated overnight at 4 °C under agitation with antibodies listed in Supplementary Table S2. The next day, membranes were washed with TBST and incubated for 1 h with the corresponding secondary HRP-conjugated goat anti-rabbit or anti-mouse IgG antibody (Jackson Immunoresearch). Proteins were visualized with an enhanced Clarity Western ECL Substrate (Bio-Rad, Hercules, CA, USA) using the ChemiDoc MP Imaging System (Bio-Rad, Hercules, CA, USA). Protein expression was measured by quantifying the density of immunoblots bands calculated relative to GAPDH using ImageJ^®^ (NIH Image, Bethesda, MD, USA), and expressed as arbitrary units (AU).

### 4.10. Scratch Wound Assay

HACs were seeded (P2) at a density of 20,000 cells/cm^2^ in 96-well ImageLock plates (Essen BioScience, Michigan, USA) and were cultured for five days to form a confluent monolayer. A WoundMakerTM (Essen BioScience, MI, USA) was used to create uniform and reproducible wounds in all wells (four wells per condition) according to the manufacturer’s instructions. After two washes with preheated PBS, Promerim^®^ hydrolysates were added with or without IL-1β (10 ng/mL) in the presence of FCS (2%), and the wound areas were monitored until 120 h, using the IncuCyte ZOOM living cell imaging system (Essen BioScience, MI, USA). The wound healing area was measured using ImageJ^®^ software by subtracting the area occupied by the cells from the initial wound surface.

### 4.11. Statistical Analysis

All experiments were repeated at least three times with HACs from different patients. Values are presented as mean ± SD or as boxplots. Statistical analyses were performed using the Mann–Whitney U-test to determine significant differences between two groups, or using a two-way ANOVA followed by a Bonferonni test for multiple comparisons. Statistical analyses were done using Prism (Graphpad, San Diego, CA, USA). A *p*-value of ≤0.05 was considered to be significant.

## Data Availability

The data presented in this study are available in Supplementary Materials.

## Supplementary Materials

The following are available online at https://www.mdpi.com/article/10.3390/ijms22073693/s1.
