# Supplementary material for: Marine Collagen Hydrolysates Promote Collagen Synthesis, Viability and Proliferation While Downregulating the Synthesis of Pro-Catabolic Markers in Human Articular Chondrocytes
Source: Int J Mol Sci. 2021 Apr 1;22(7):3693. doi: 10.3390/ijms22073693 (PMC8036580; doi:10.3390/ijms22073693)
Supplement: Supplementary file 1 [file ijms-22-03693-s001.zip › Supplementary information Bourdon et al. 03-31-2021.pdf]

Marine Collagen Hydrolysates Promote Collagen Synthesis, Viability and Proliferation While Downregulating the Synthesis of Pro-Catabolic Markers in Human Articular Chondrocytes

Bastien Bourdon [1,2], Frédéric Cassé [1], Nicolas Gruchy [1,3], Pierre Cambier [1], Sylvain Leclercq [4], Sarah Oddoux [2], Antoine Noël [2], Jérôme Lafont [5], Romain Contentin [1,†] and Philippe Galéra [1,*,†]

1 BIOTARGEN, Normandie University, UNICAEN 14000 Caen, France; bastien.bourdon@unicaen.fr (B.B.); frederic.casse@unicaen.fr (F.C.); nicolas.gruchy@unicaen.fr (N.G.); 21507655@etu.unicaen.fr (P.C.); contentinr@email.chop.edu (R.C.); philippe.galera@unicaen.fr (P.G.)

2 Dielen laboratory, 50110 Tourlaville, France; sarah-oddoux@dielen.fr (S.O.); antoine-noel@dielen.fr (A.N.)

3 Department of Genetics, Normandy Center for Genomic and Personalized Medicine, Caen University Hospital, 14000 Caen, France

4 Service de Chirurgie Orthopédique, Clinique Saint-Martin, 14000 Caen, France ; sylvainleclercq60@gmail.com

5 CNRS UMR 5305, Laboratory of Tissue Biology and Therapeutic Engineering, University Claude Bernard Lyon 1, Univ Lyon, 69367 Lyon, France; jerome.lafont@ibcp.fr

* Correspondence: philippe.galera@unicaen.fr

† These authors contributed equally to this work.

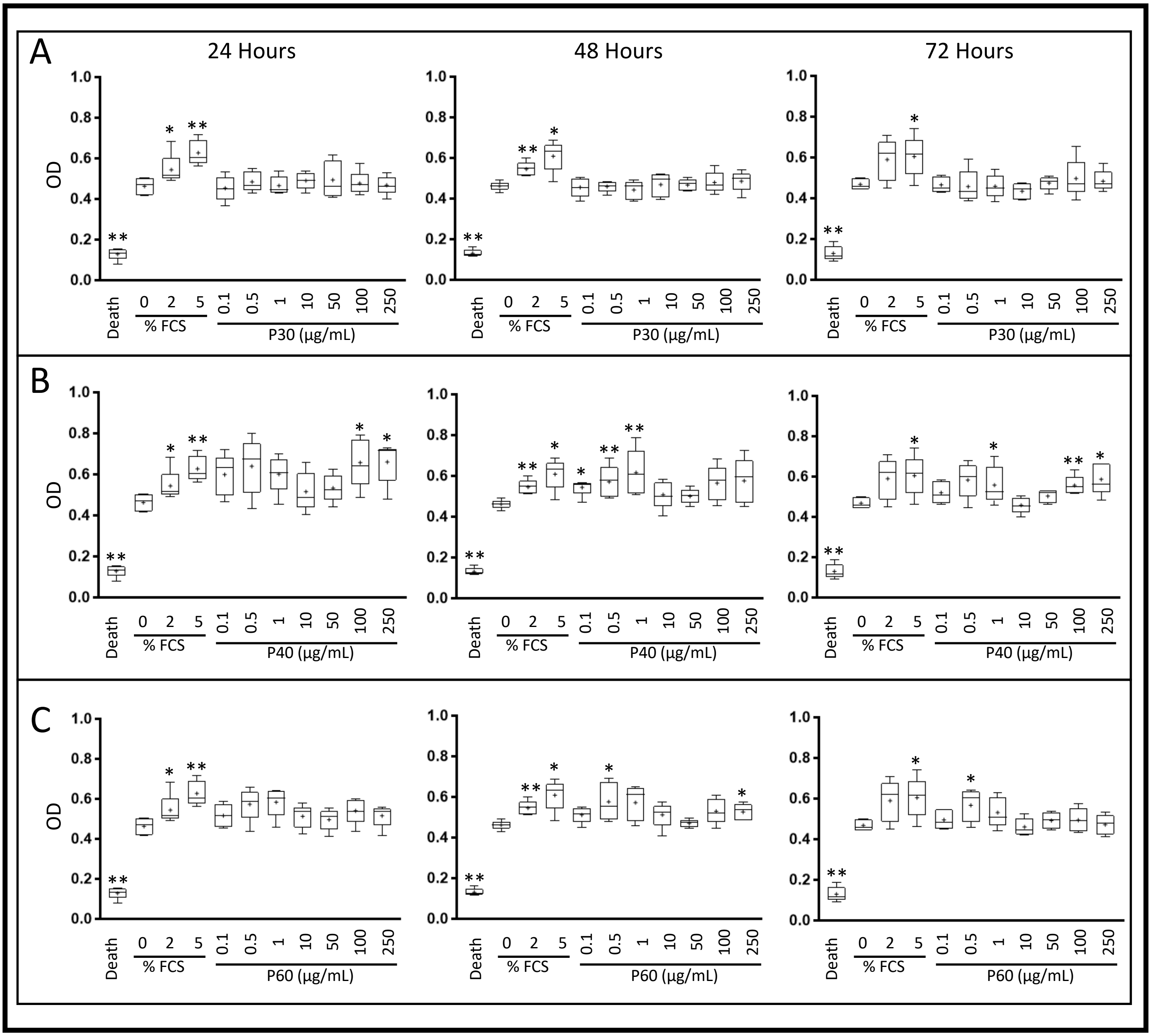

Figure S1: Effect of Promerim® hydrolysates on the viability and prolideration of human articular chondrocytes.

Human articular chondrocytes at P2 and 80% confluency were treated in the absence of FCS, with 0.1, 0.5, 1, 10, 50, 100 or 250 µg/mL Promerim®30 (A), 40 (B) or 60 (C) for 24, 48 and 72 hours in normoxia. Controls with 0%, 2% and 5% FCS, and a positive control of 100% cell death (Triton-induced death) were performed. Formazan levels, which are correlated with viability and proliferation, were measured in the media after 24, 48 and 72 hours of treatment. Results are summarized in box-plots (n= 5) showing the mean (cross) and the median (line) of optical density (OD). Statistical analyses were performed using the Mann-Whitney test (* $p<0.05$; ** $p<0.01$) compared with the 0% FCS condition.

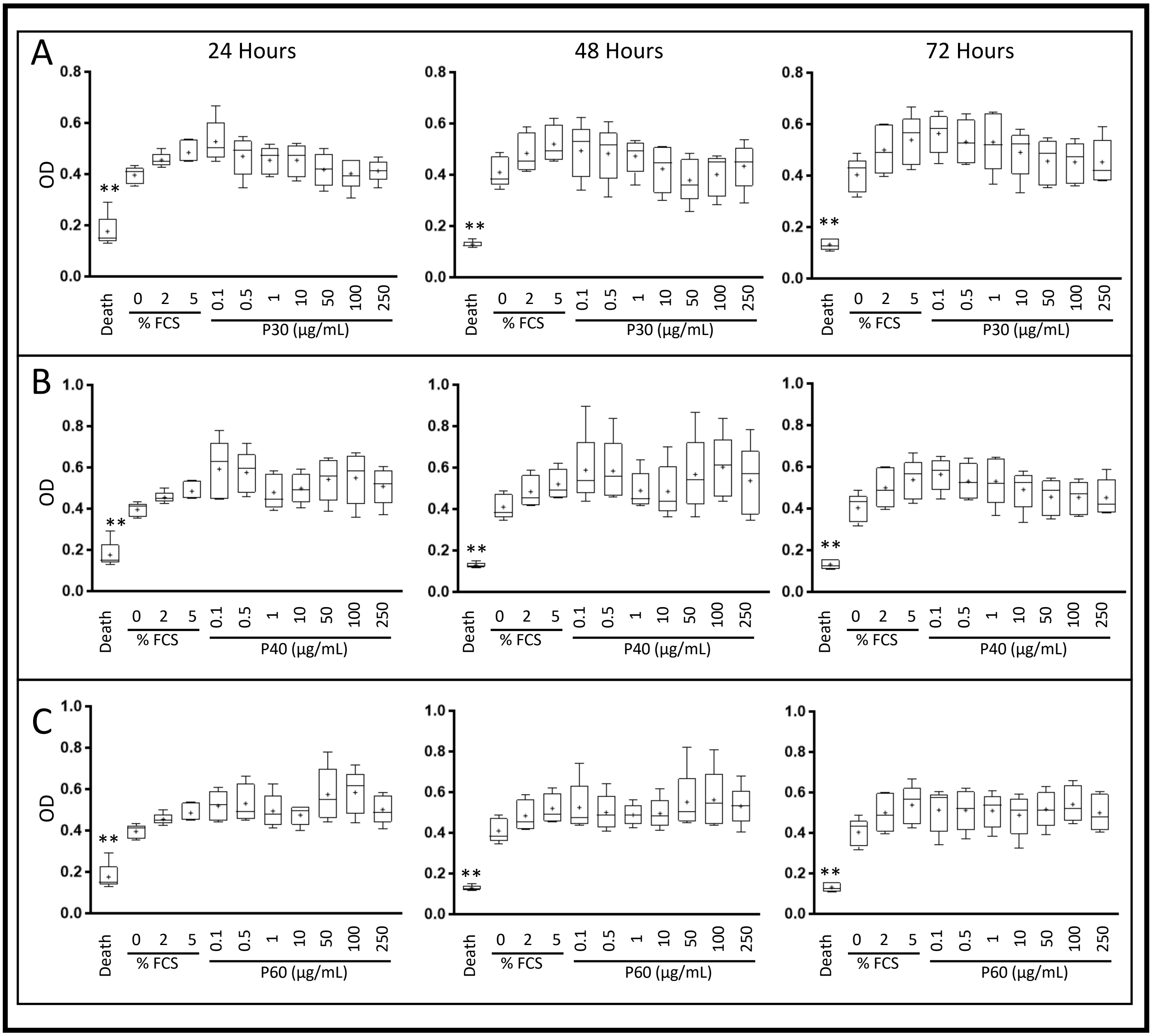

Figure S2: Effect of Promerim® hydrolysates on the viability and proliferation of human articular chondrocytes.

Human articular chondrocytes at P2 and 80% confluency were treated in the presence of 2% FCS, with 0.1, 0.5, 1, 10, 50, 100 or 250 µg/mL Promerims®30 (A), 40 (B) and 60 (C) for 24, 48 and 72 hours in normoxia. Controls with 0%, 2% and 5% FCS, and a positive control of 100% cell death (Triton-induced death) were performed. Formazan levels, which are correlated with viability and proliferation, were measured in the media after 24, 48 and 72 hours of treatment. Results are summarized in box-plots (n= 5) showing the mean (cross) and the median (line) of optical density (OD). Statistical analyses were performed using the Mann-Whitney test (** $p<0.01$) compared with the 2% FCS condition.

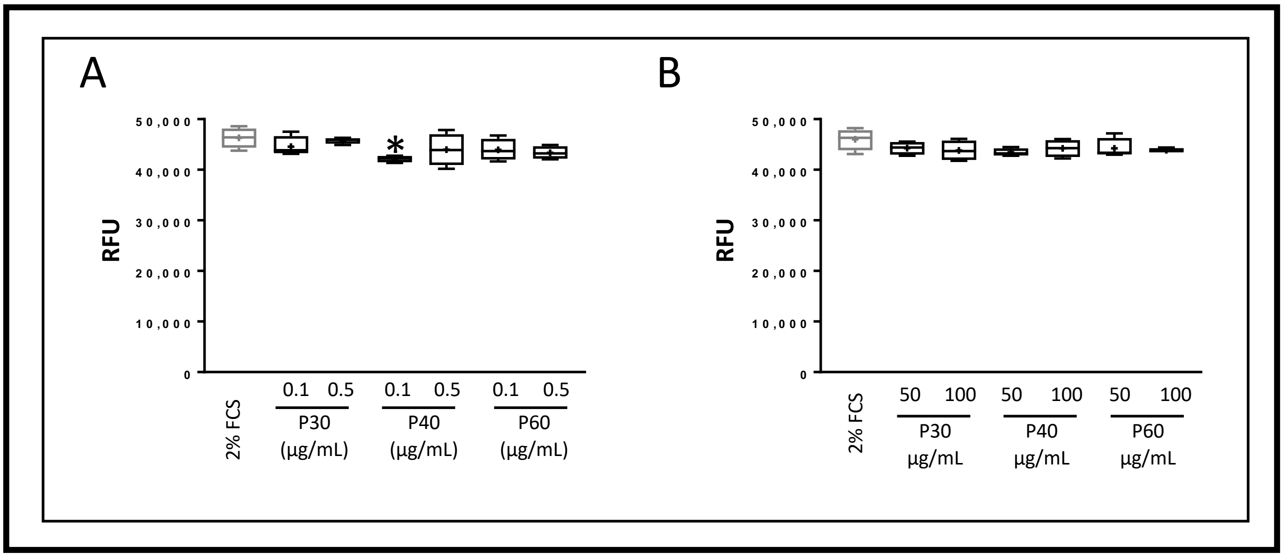

Figure S3: Effect of Promerim® 30, 40 and 60 on the senescence of human articular chondrocytes. Human articular chondrocytes at P2 and 80% confluency were treated in the presence of 2% FCS, with Promerim® hydrolysates (P30, P40 and P60) at 0.1 and 0.5 µg/mL (A), 50 and 100 µg/mL (B) for 72 hours in normoxia. Controls with 2% FCS were performed (Ctrl) and the levels of β-galactosidase were measured 3 days post-treatment. Results are summarized in box-plots (n= 4) showing the mean (cross). Statistical analyses were performed using the Mann-Whitney test (* $p<0.05$) and the control condition was used as the reference.

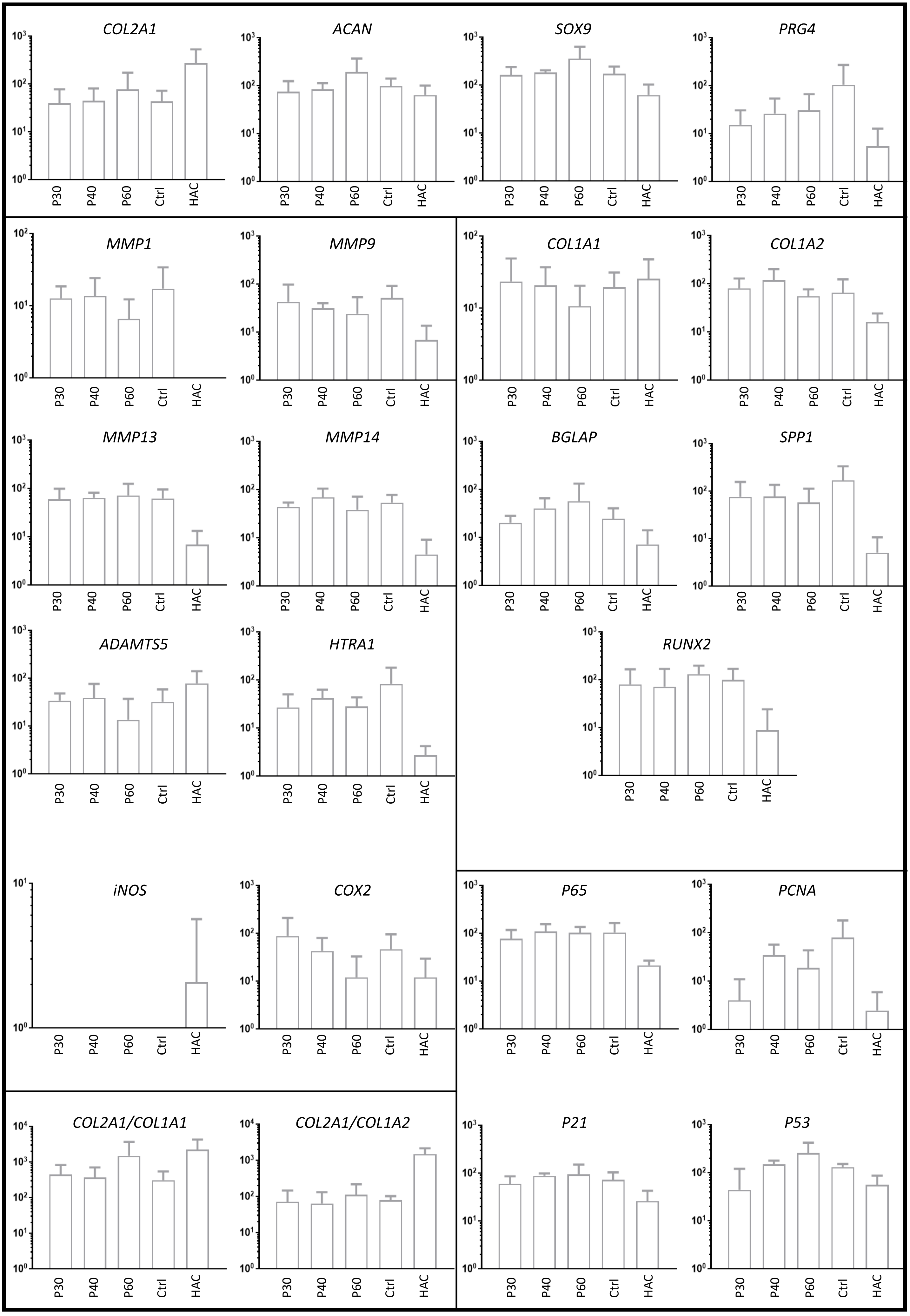
COL2A1
ACAN
SOX9
PRG4
MMP1
MMP9
COL1A1
COL1A2
MMP13
MMP14
BGLAP
SPP1
ADAMTS5
HTRA1
RUNX2
iNOS
COX2
P65
PCNA
COL2A1/COL1A1
COL2A1/COL1A2
P21
P53
P30
P40
P60
Ctrl
HAC

Figure S4: Effect of low concentrations of Promerim®30, 40 and 60 on gene expression profile in human articular chondrocytes. Human articular chondrocytes were grown in type I/III collagen sponges at (P2) for 7 days in hypoxia in the absence (Ctrl: control) or presence of Promerim®30, 40 or 60 at 0.1 µg/mL (P30, P40 and P60). Results are represented as histograms (n= 3) showing the mean ± SE of relative mRNA expression estimated using RT-qPCR and normalized to the *β-ACTIN* and *PPIA* reference gene. The *COL2A1*:*COL1A1* and *COL2A1*:*COL1A2* ratios are given. HAC: mRNA obtained from human articular chondrocytes at P0 were used as controls. Statistical analyses were performed using the Mann-Whitney test compared with the control condition.

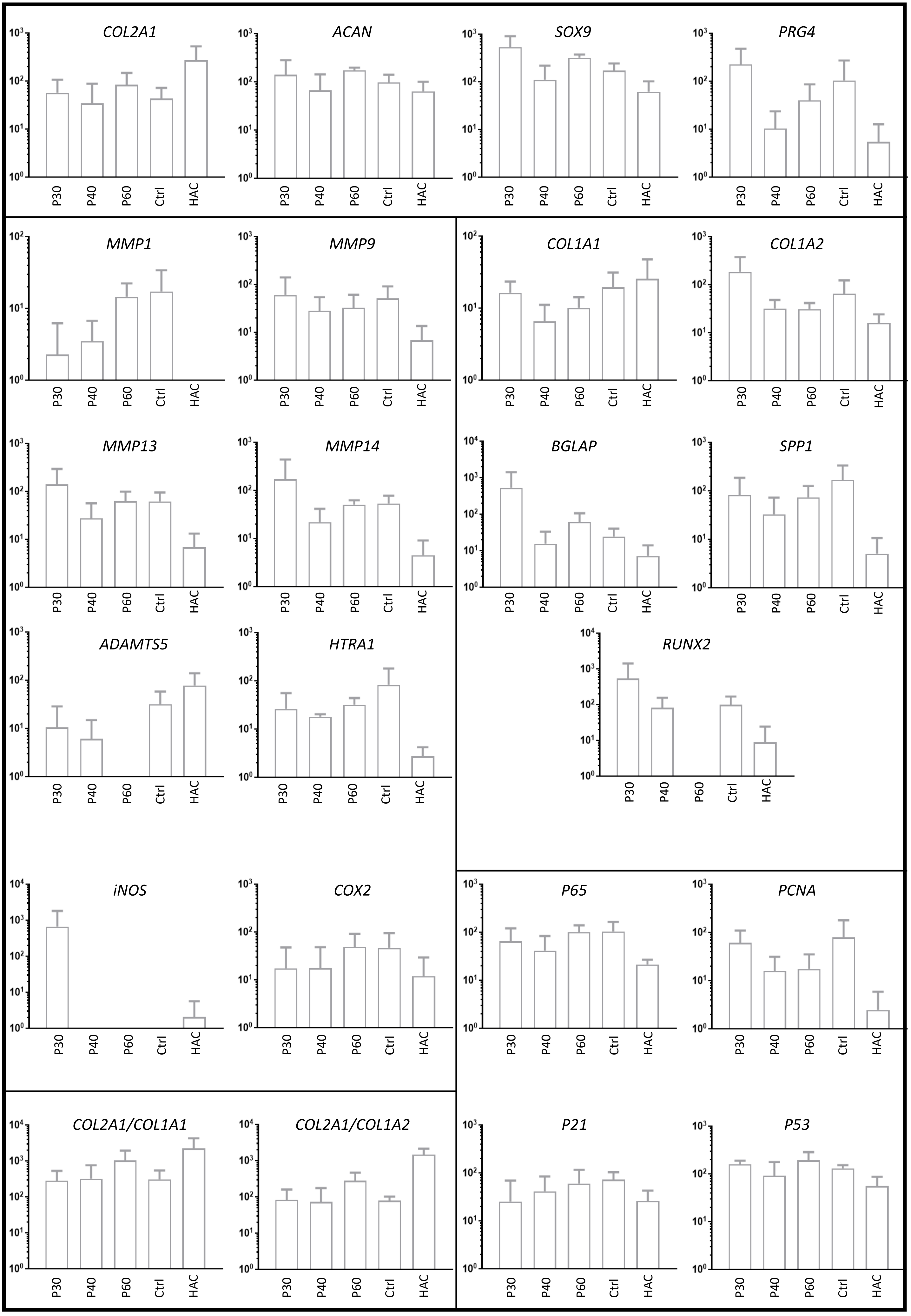
COL2A1
ACAN
SOX9
PRG4
MMP1
MMP9
COL1A1
COL1A2
MMP13
MMP14
BGLAP
SPP1
ADAMTS5
HTRA1
RUNX2
iNOS
COX2
P65
PCNA
COL2A1/COL1A1
COL2A1/COL1A2
P21
P53
P30
P40
P60
Ctrl
HAC

Figure S5: Effect of low concentrations of Promerim®30, 40 and 60 on gene expression profile in human articular chondrocytes. Human articular chondrocytes were grown in type I/III collagen sponges at (P2) for 7 days in hypoxia in the absence (Ctrl: control) or presence of Promerim®30, 40 or 60 at 0.5 µg/mL (P30, P40 and P60). Results are represented as histograms (n= 3) showing the means ± SE of relative mRNA expression estimated using RT-qPCR and normalized to the *β-ACTIN* and *PPIA* reference gene. The *COL2A1*:*COL1A1* and *COL2A1*:*COL1A2* ratios are given. HAC: mRNA obtained from human articular chondrocytes at P0 were used as controls. Statistical analyses were performed using the Mann-Whitney test compared with the control condition.

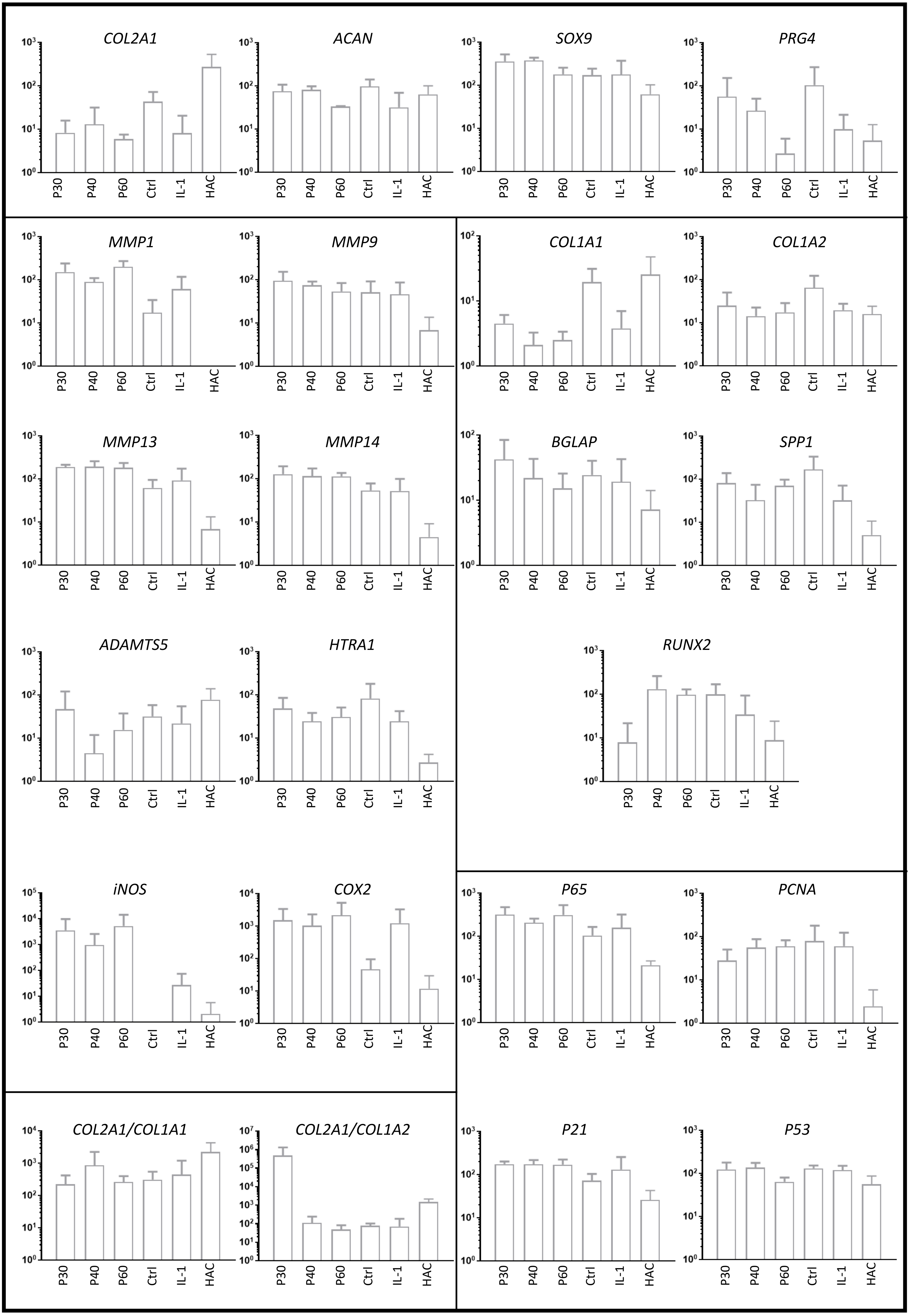
COL2A1
ACAN
SOX9
PRG4
MMP1
MMP9
COL1A1
COL1A2
MMP13
MMP14
BGLAP
SPP1
ADAMTS5
HTRA1
RUNX2
iNOS
COX2
P65
PCNA
COL2A1/COL1A1
COL2A1/COL1A2
P21
P53
P30
P40
P60
Ctrl
IL-1
HAC
10^0
10^1
10^2
10^3
10^4
10^5
10^6
10^7

Figure S6: Effect of low concentrations of Promerim®30, 40 and 60 on gene expression profile in human articular chondrocytes. Human articular chondrocytes were grown in type I/III collagen sponges at (P2) for 7 days in hypoxia, treated with IL-1β (10 ng/mL) or without (Ctrl: control) and/or the presence of Promerim®30, 40 or 60 at 0.1 µg/mL (P30, P40 and P60). Results are represented as histograms (n= 3) showing the means ± SE of relative mRNA expression estimated using RT-qPCR and normalized to the *β-ACTIN* and *PPIA* reference gene. The *COL2A1*:*COL1A1* and *COL2A1*:*COL1A2* ratios are given. HAC: mRNA obtained from human articular chondrocytes at P0 were used as controls. Statistical analyses were performed using the Mann-Whitney test compared with the IL-1 condition.

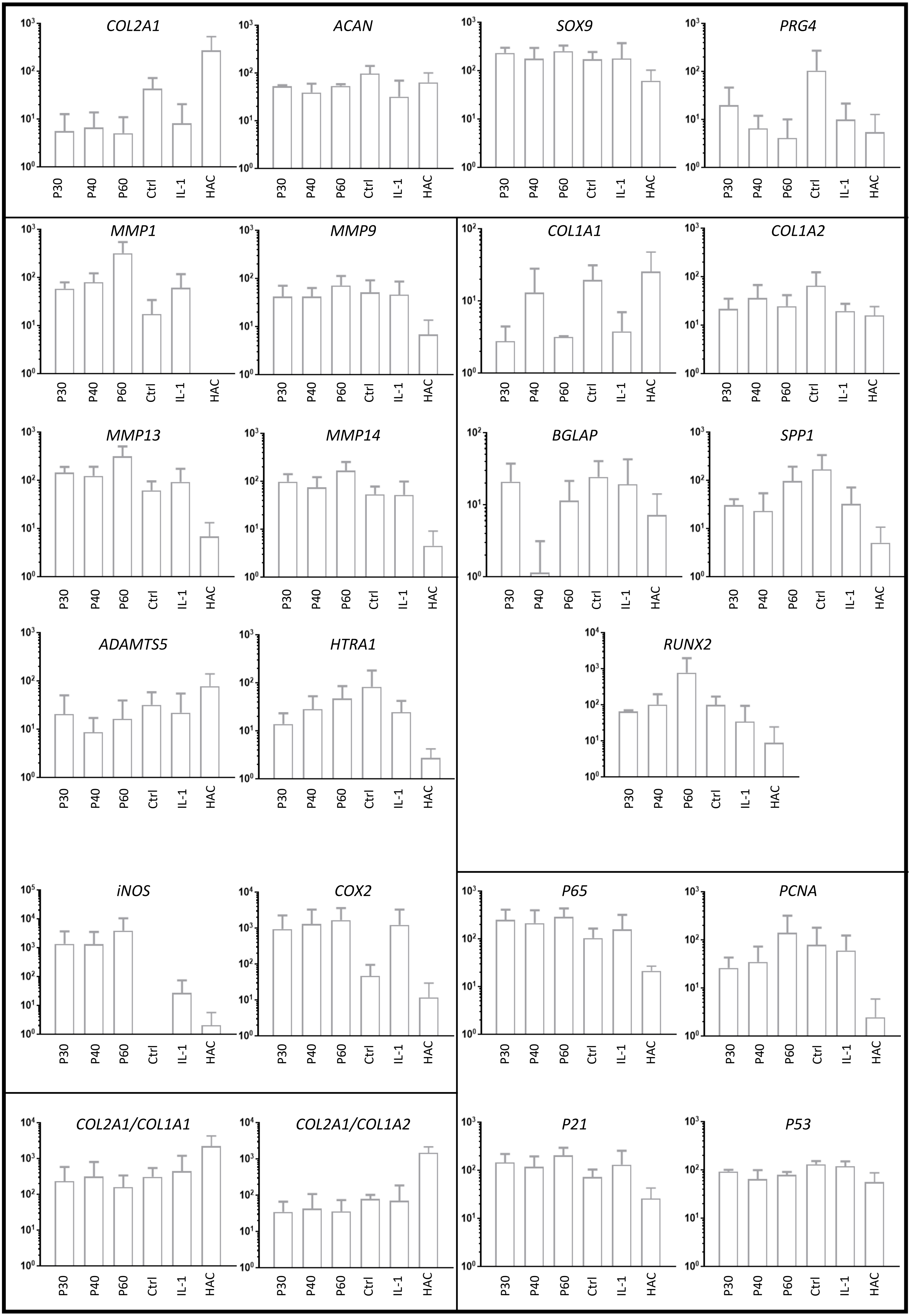
COL2A1
ACAN
SOX9
PRG4
MMP1
MMP9
COL1A1
COL1A2
MMP13
MMP14
BGLAP
SPP1
ADAMTS5
HTRA1
RUNX2
iNOS
COX2
P65
PCNA
COL2A1/COL1A1
COL2A1/COL1A2
P21
P53
P30
P40
P60
Ctrl
IL-1
HAC

Figure S7: Effect of low concentrations of Promerim®30, 40 and 60 on gene expression profile in human articular chondrocytes. Human articular chondrocytes were grown in type I/III collagen sponges at (P2) for 7 days in hypoxia, treated with IL-1β (10 g/mL) or not (Ctrl: control) and/or the presence of Promerim®30, 40 or 60 at 0.5 µg/mL (P30, P40 and P60). Results are represented as histograms (n= 3) showing the mean ± SE of relative mRNA expression estimated using RT-qPCR and normalized to the *β-ACTIN* and *PPIA* reference gene. The *COL2A1*:*COL1A1* and *COL2A1*:*COL1A2* ratios are given. HAC: mRNA obtained from human articular chondrocytes at P0 were used as controls. Statistical analyses were performed using the Mann-Whitney test compared with the IL-1 condition.

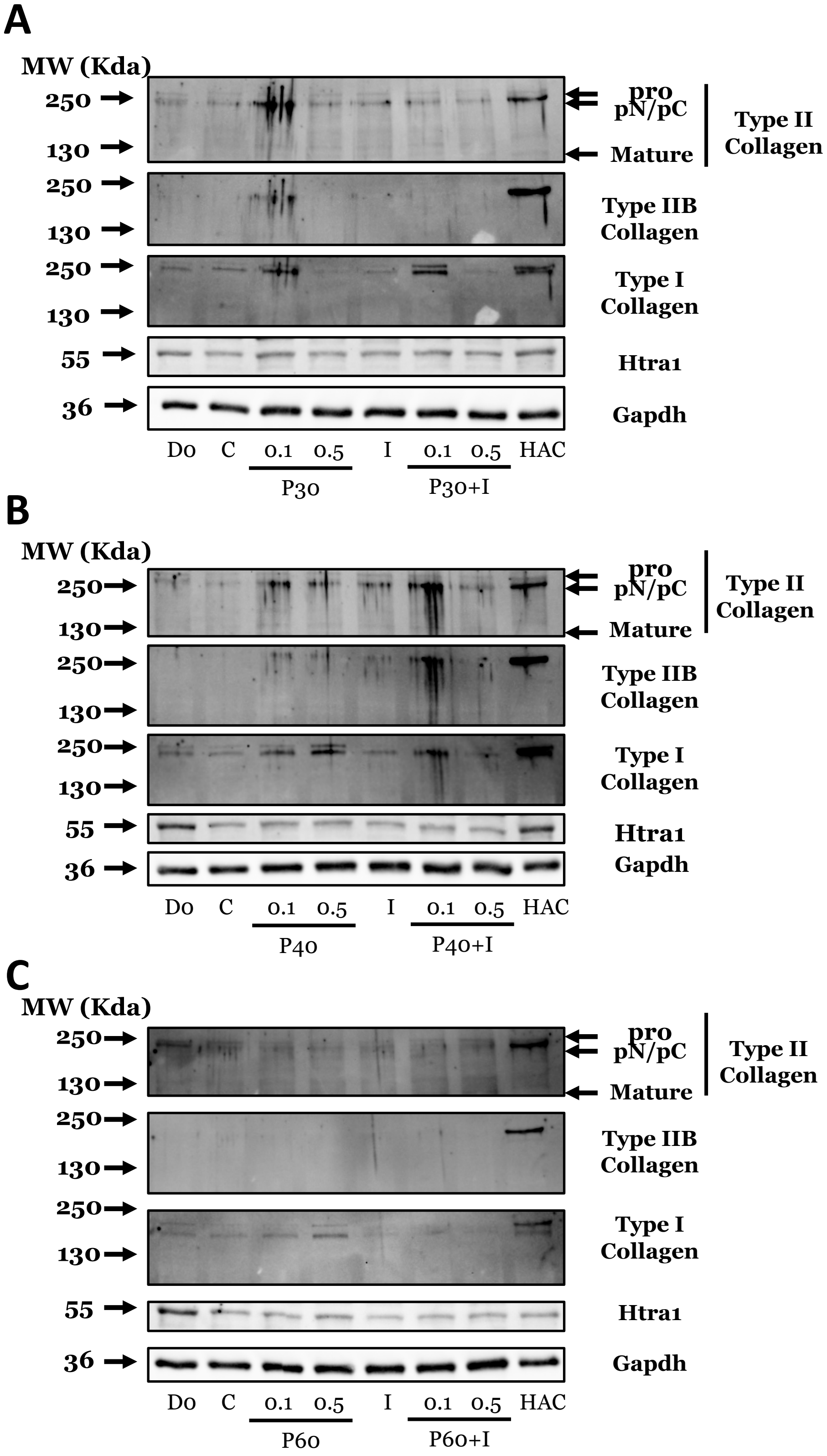

Figure S8: Effect of low concentrations of Promerim® on collagens and HtrA1 protein expression of human articular chondrocytes. Human articular chondrocytes at P2 were inoculated in collagen sponges and treated for 7 days in the absence or presence of IL-1β (10 ng/mL) with Promerim®30 (A), 40 (B) or 60 (C) at 0.1 and 0.5 µg/mL. The 3D control culture medium was used as control (C). I (IL-1), P30 (Promerim®30), P40 (Promerim®40), P60 (Promerim®60). Cells seeded in sponges stopped at day 0 (D0) and protein extracts obtained from human articular cartilage (HAC). The molecular weights expressed in kDa are shown on the left-hand side of the panels and the target proteins on the right-hand side of the panels. Images show representative immunoblots from different HAC strains (at least n= 3). Full membranes are shown in figure S14 as well as under-exposed and over-exposed blots in figures S15 and S16, respectively.

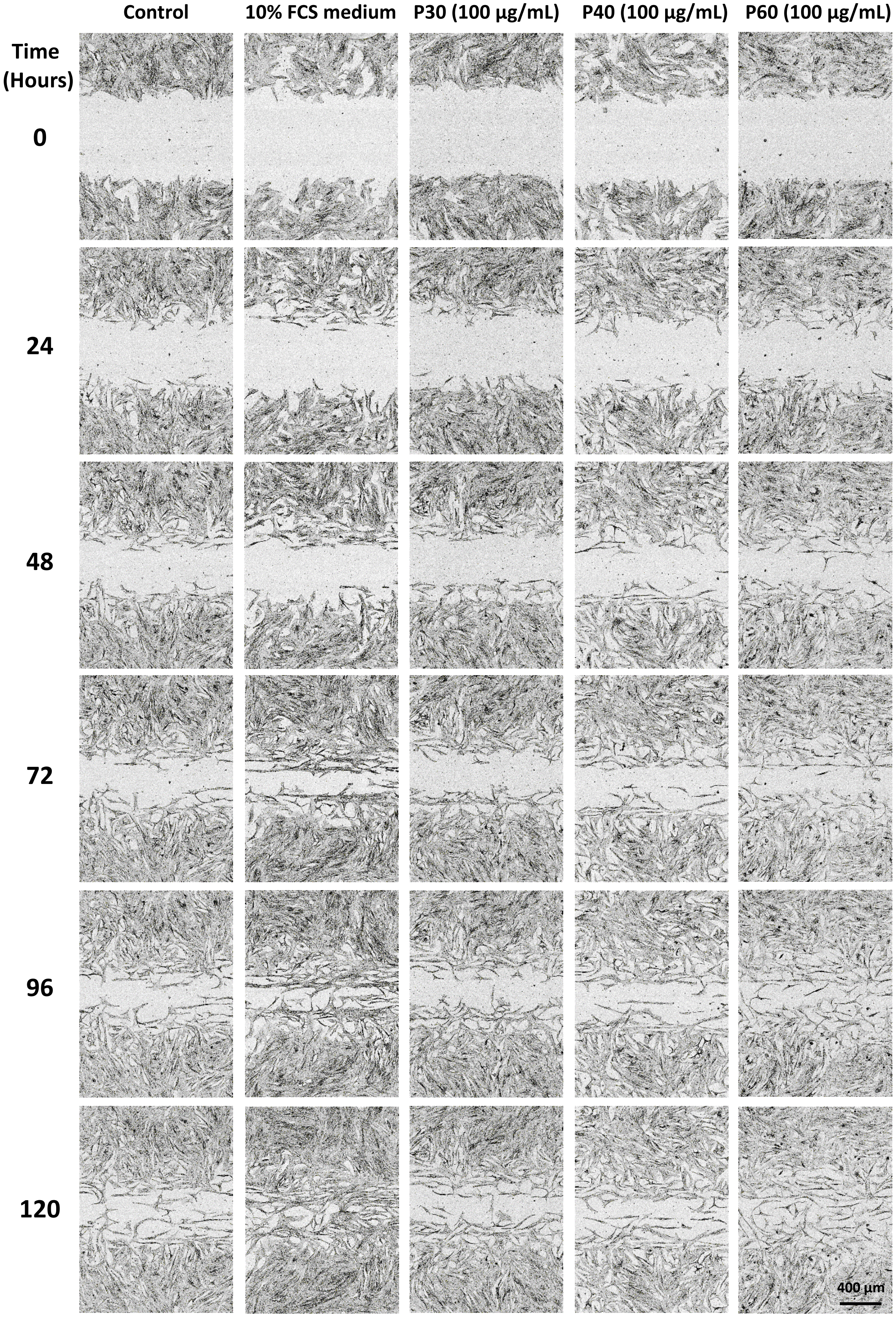

Figure S9: Microscopy of wound filling analysis following a scratch wound assay. Microscopy of human articular chondrocytes seeded at P2. At 90% confluency, a scratch wound assay was performed using a WoundMakerTM kit (Essen BioScience) and then the treatments were added. HACs were incubated in the presence of a culture medium containing 2% FCS (Control), and P30, P40, or P60 at 50 and 100 µg/mL. P30, P40 and P60: Promerim®30, Promerim®40 and Promerim®60.

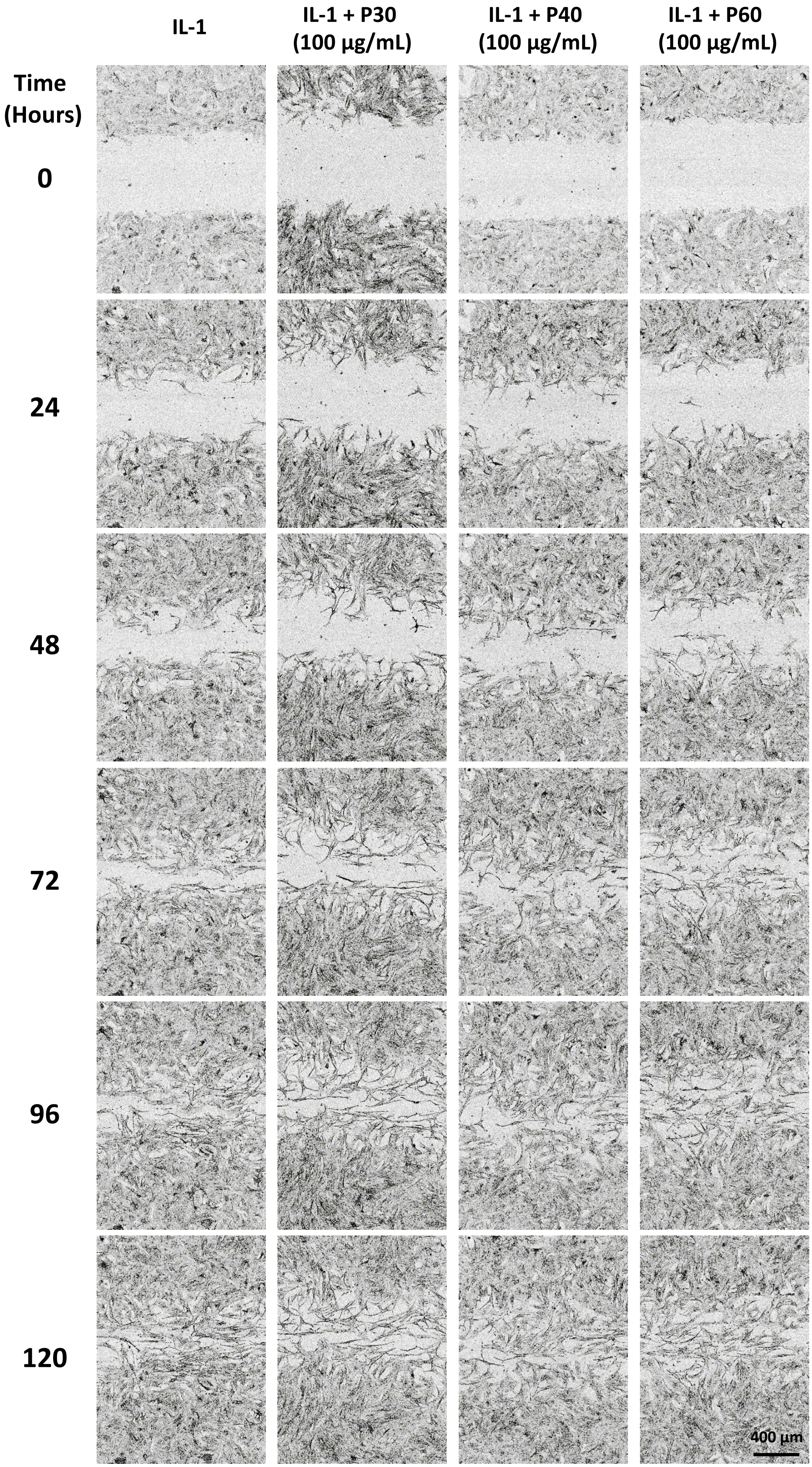

Figure S10: Microscopy of wound filling analysis following a scratch wound assay. Misroscopy of human articular chondrocytes seeded at P2. At 90% of confluency, a scratch wound assay was performed using a WoundMakerTM kit (Essen BioScience) and then the treatments were added. HACs were incubated in the presence of a culture medium containing 2% FCS (Control), and P30, P40, or P60 at 50 and 100 µg/mL with IL-1. P30, P40 and P60: Promerim®30, Promerim®40 and Promerim®60.

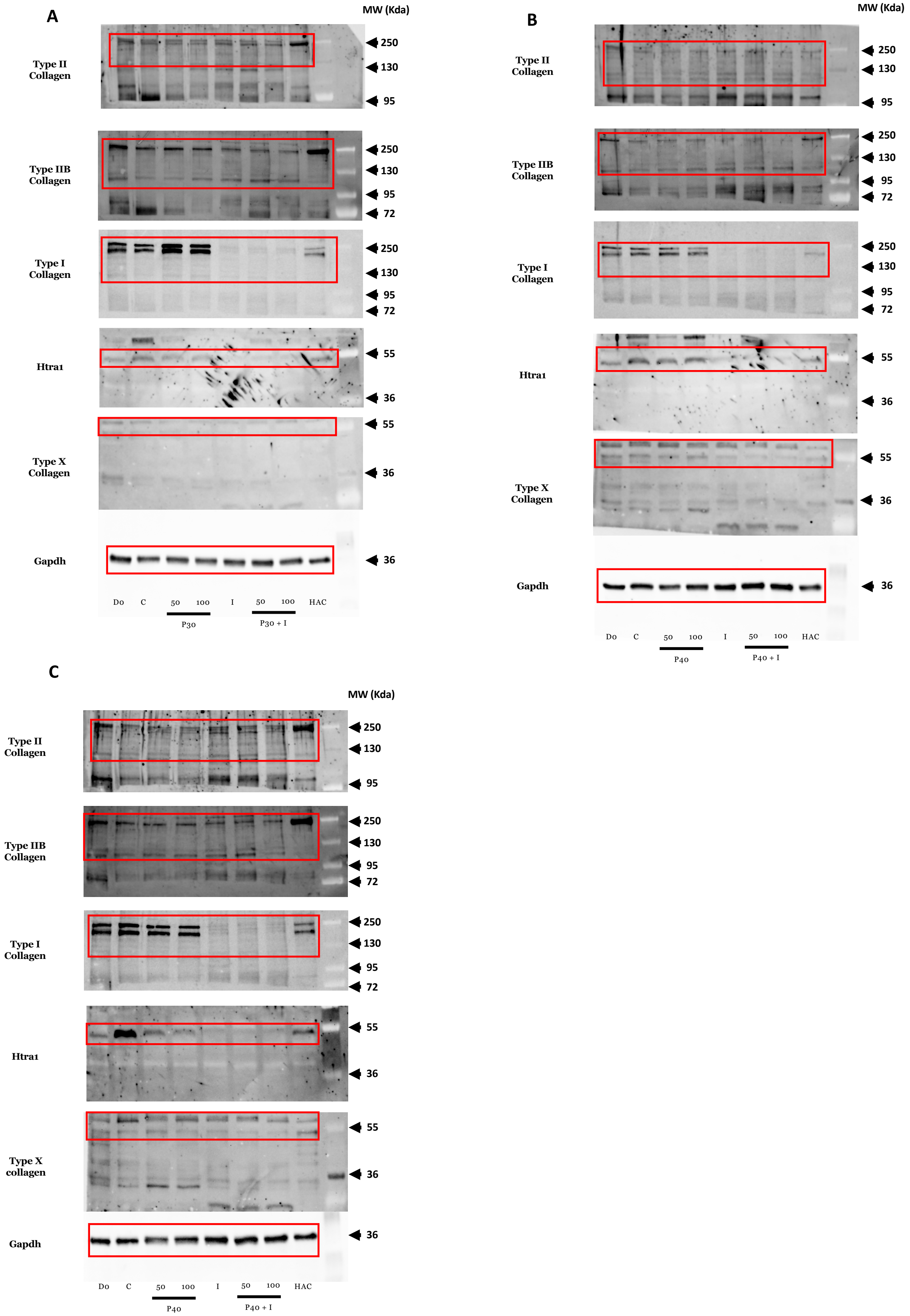

Figure S11: Complete gel and PVDF membranes analyzed in the western-blots.

For the western-blots presented in figure 10, respectively A, B and C, the complete images of the membranes captured with Chemidoc MP Imaging System (Bio-Rad) are shown. The molecular weight marker (MW kDa) is indicated on the right for the images shown. The cropped images are highlighted in the red lines.

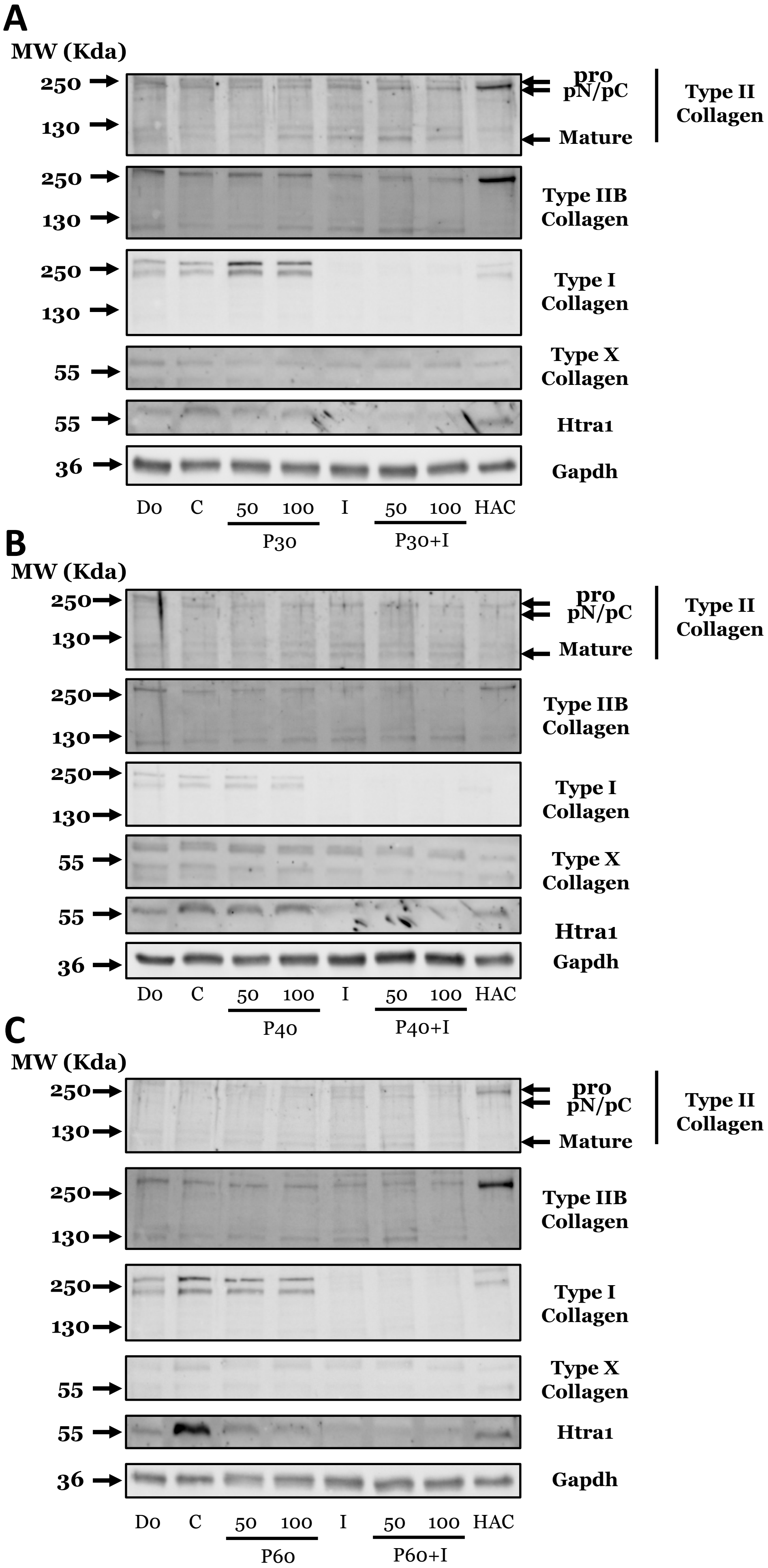

Figure S12: Under-exposure of the blots presented in figure 10.

Under-exposure (A, B, C) are shown. The molecular weight marker (MW kDa) is indicated on the left for the images presented.

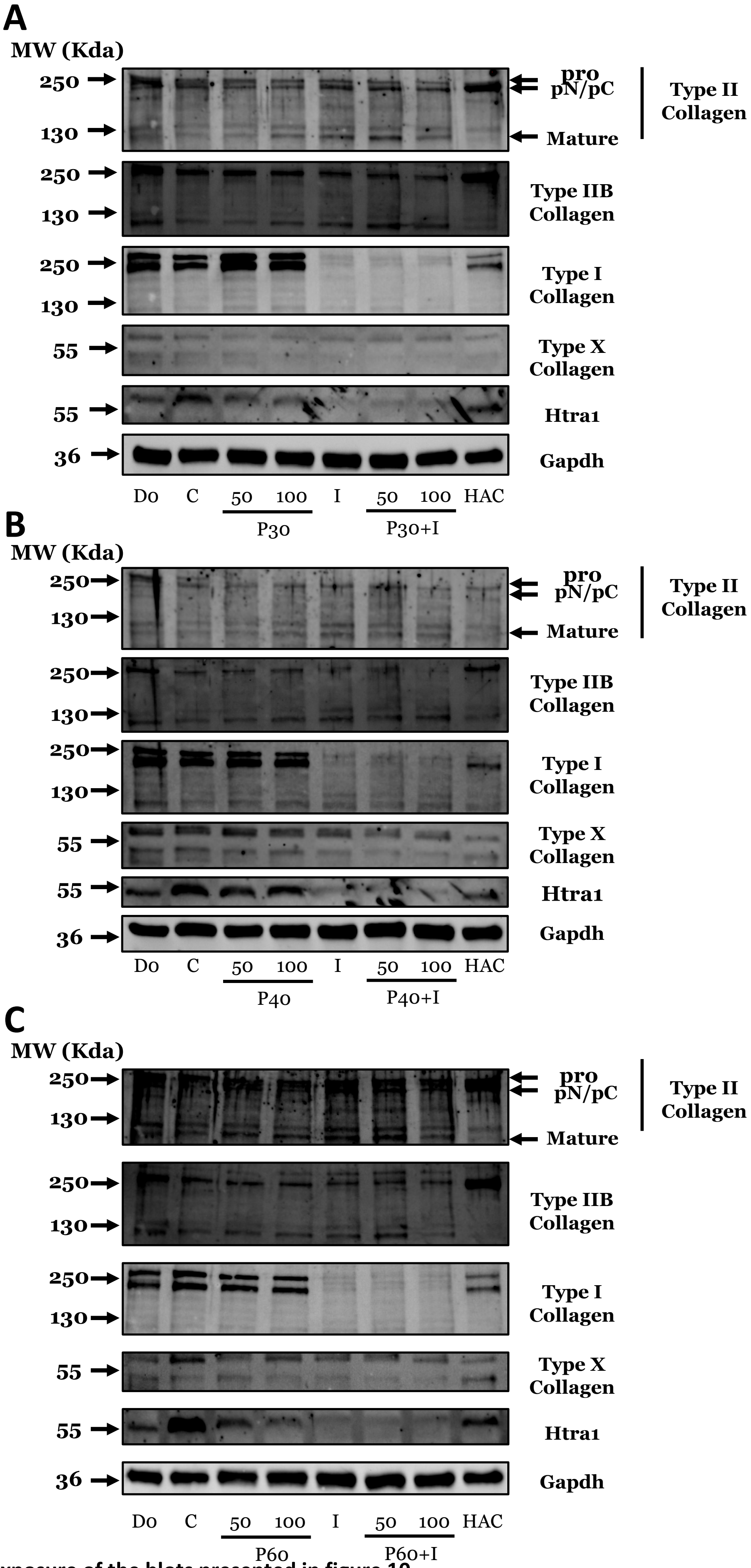

Figure S13: Over-exposure of the blots presented in figure 10.

Over-exposure (A, B, C) are shown. The molecular weight marker (MW kDa) is indicated on the left for the images presented.

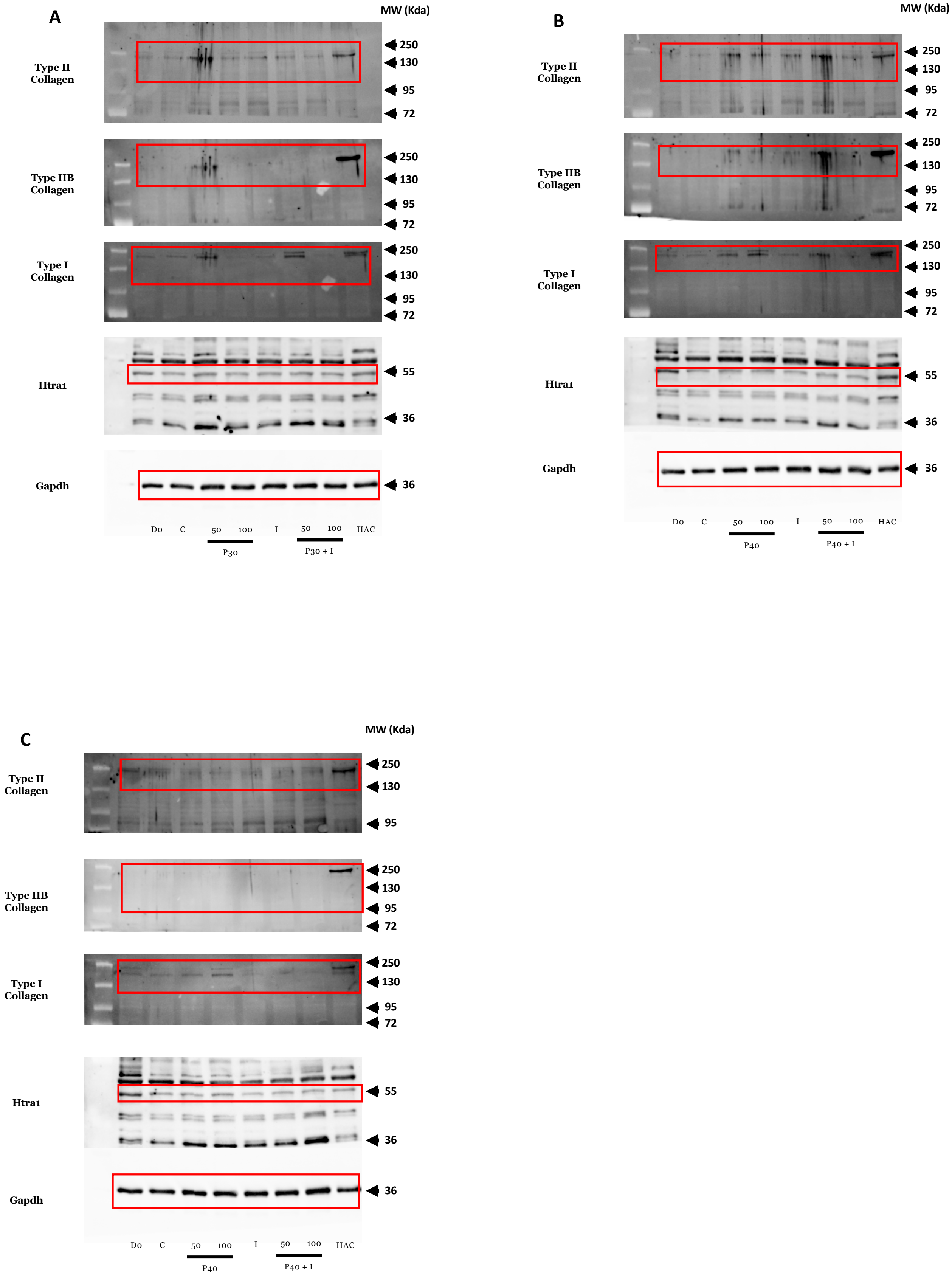

Figure S14: Complete gel and PVDF membranes analyzed in the western-blots.

For the western-blots presented in figure S8, respectively A, B and C, the complete images of the membranes captured with Chemidoc MP Imaging System (Bio-Rad) are shown. The molecular weight marker (MW kDa) is indicated on the right for the images shown. The cropped images are highlighted in the red lines.

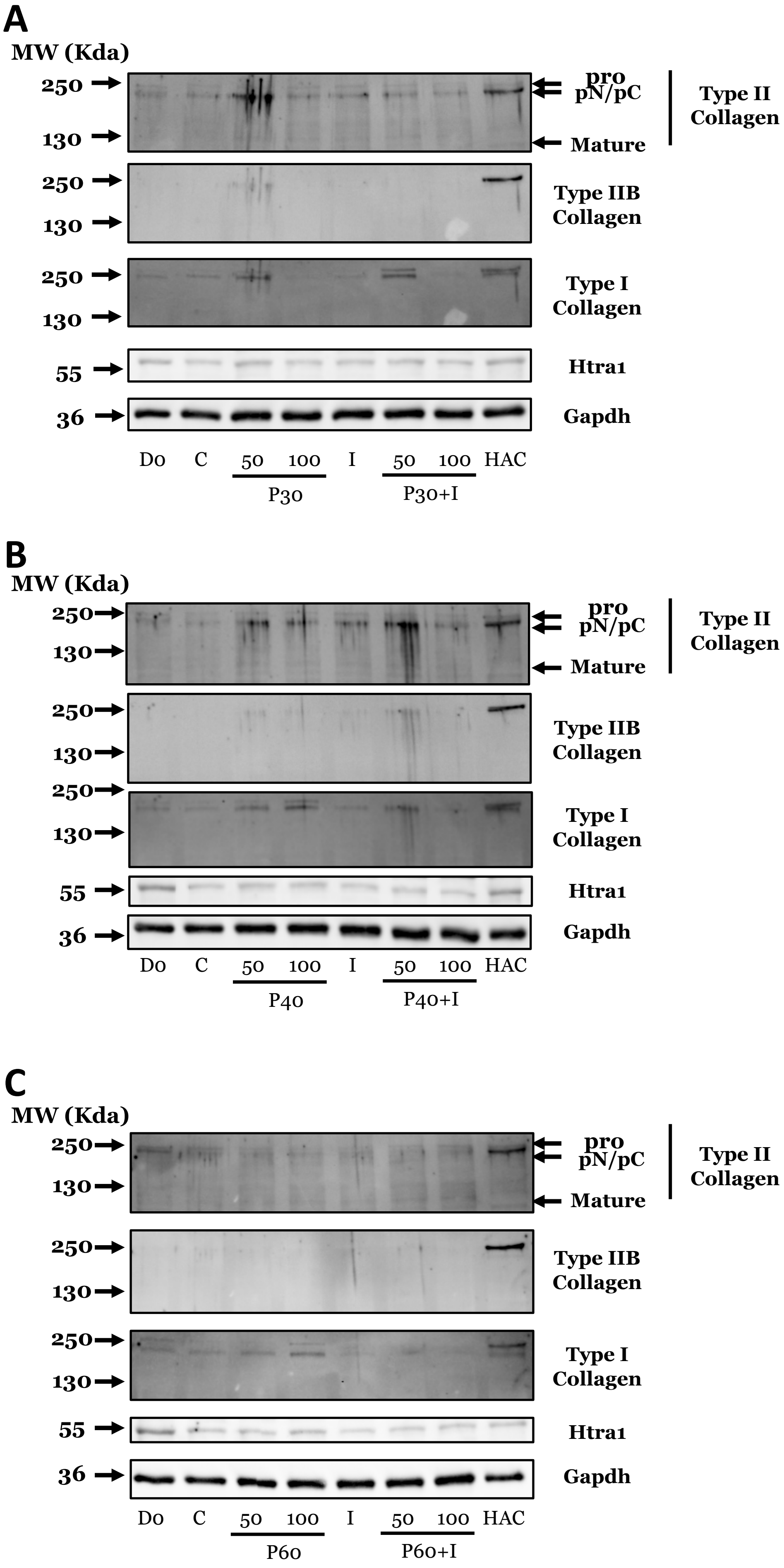

Figure S15: Under-exposure of the blots presented in figure S8.

Under-exposure (A, B, C) are shown. The molecular weight marker (MW kDa) is indicated on the left for the images presented.

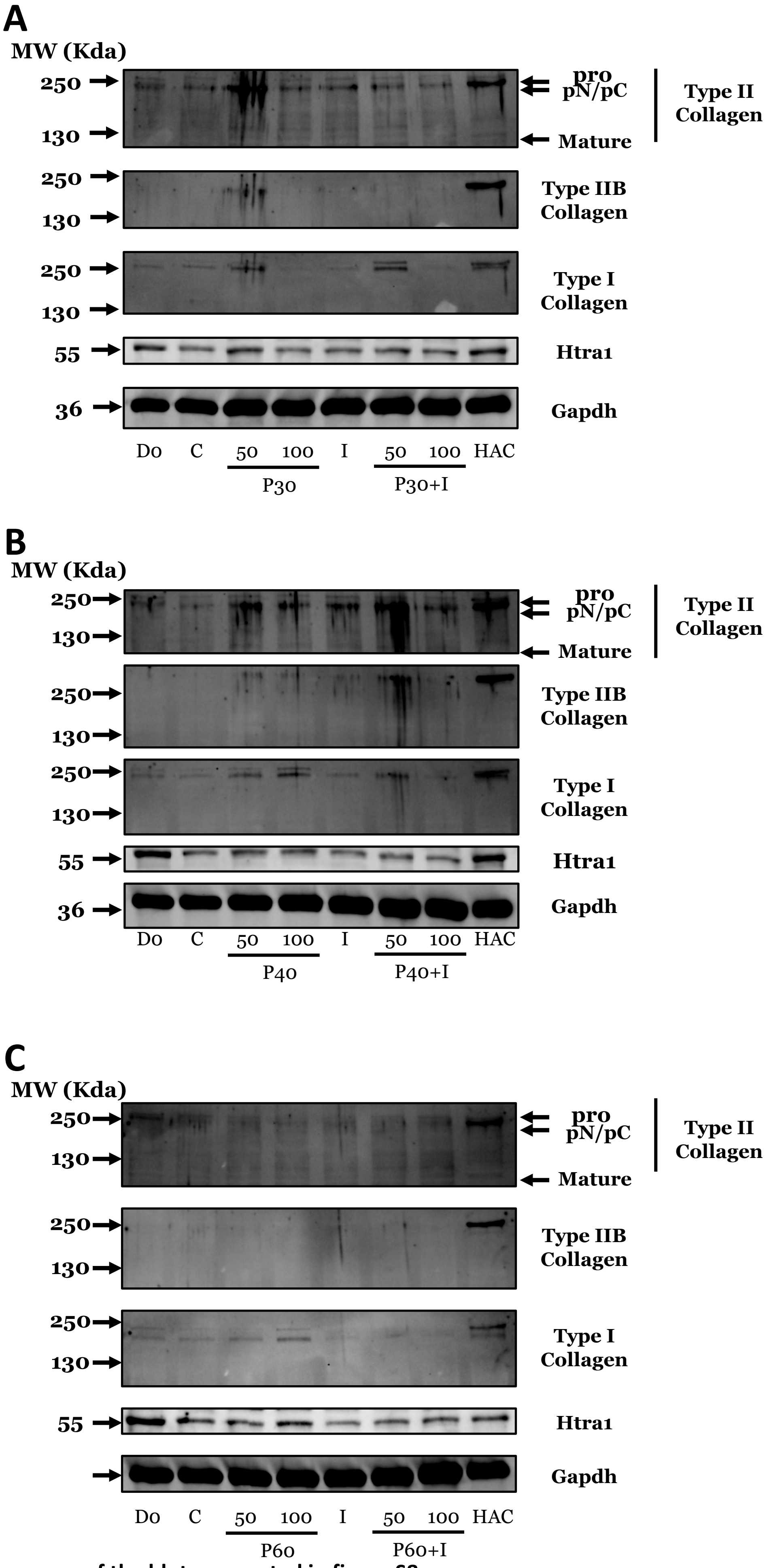

Figure S16: Over-exposure of the blots presented in figure S8.

Over-exposure (A, B, C) are shown. The molecular weight marker (MW kDa) is indicated on the left for the images presented.

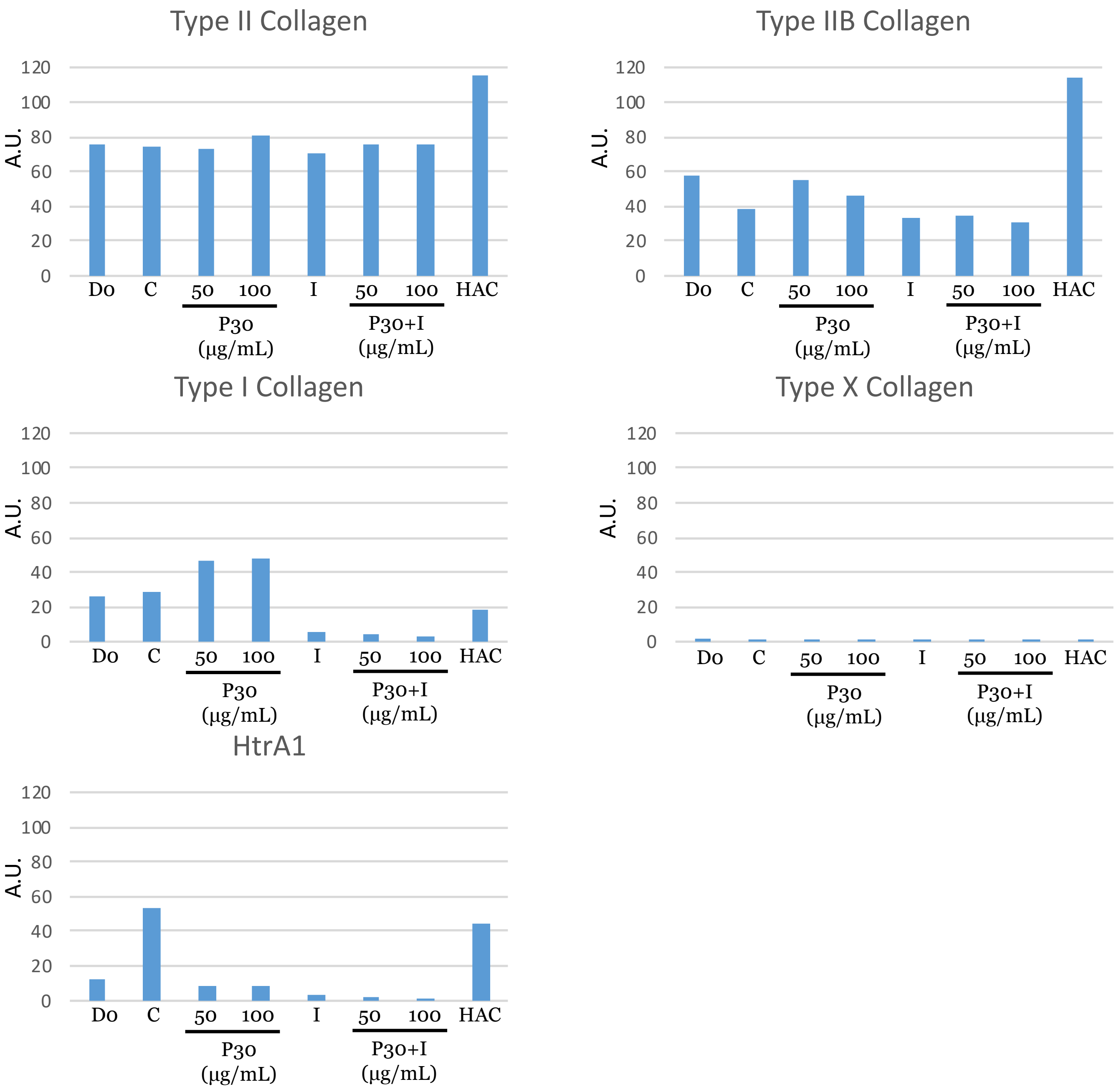

Figure S17A: Quantification of type II, IIB, I and X collagens and of Htra1 western-blots presented in Figure 10A. Protein expression was measured by quantifying the density of immunoblots bands calculated relative to GAPDH using image analysis software ImageJ®. A.U.: Arbitrary Unit.

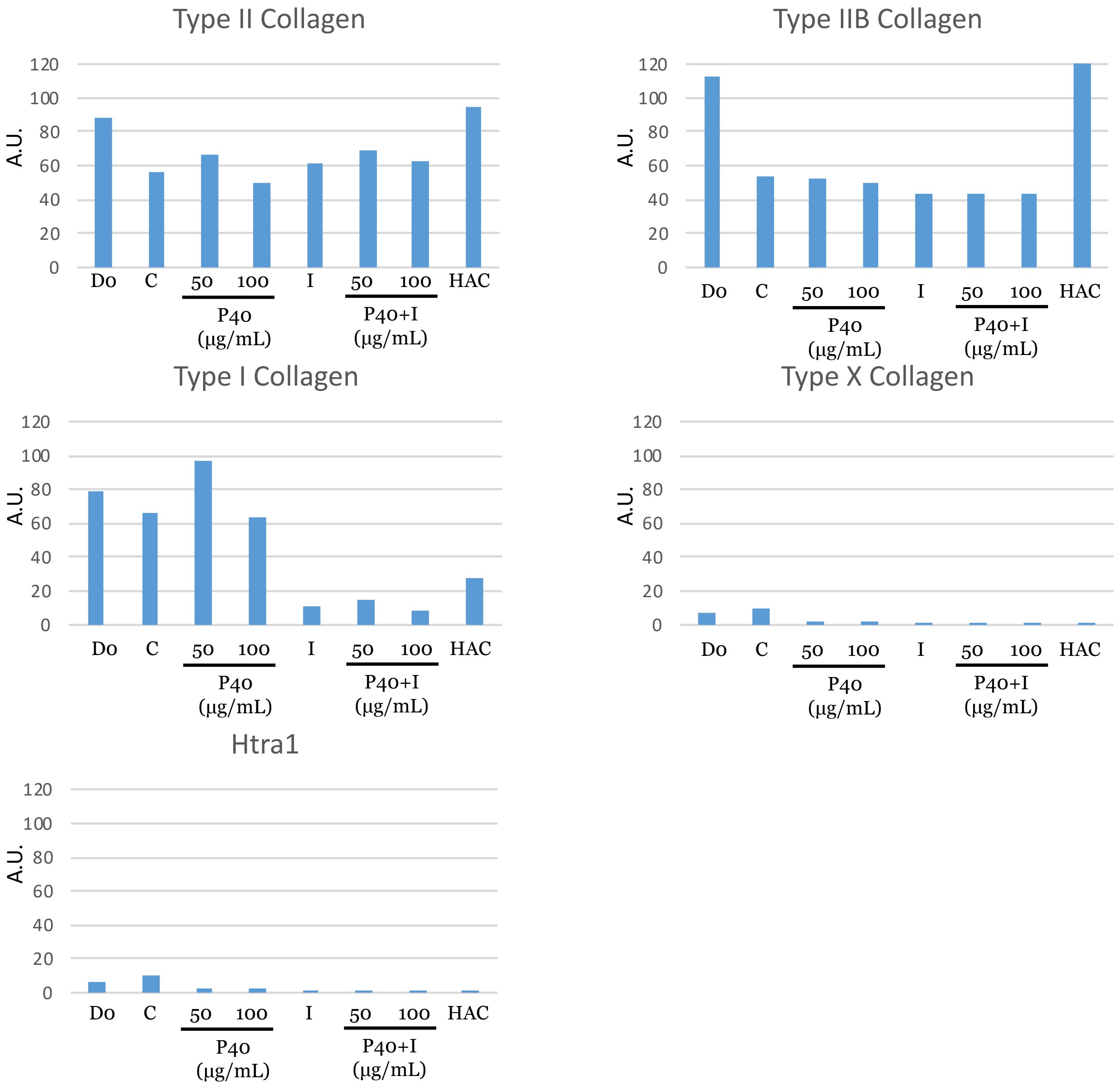

Figure S17B: Quantification of type II, IIB, I and X collagens and of Htra1 western-blots presented in Figure 10B. Protein expression was measured by quantifying the density of immunoblots bands calculated relative to GAPDH using image analysis software ImageJ®. A.U.: Arbitrary Unit.

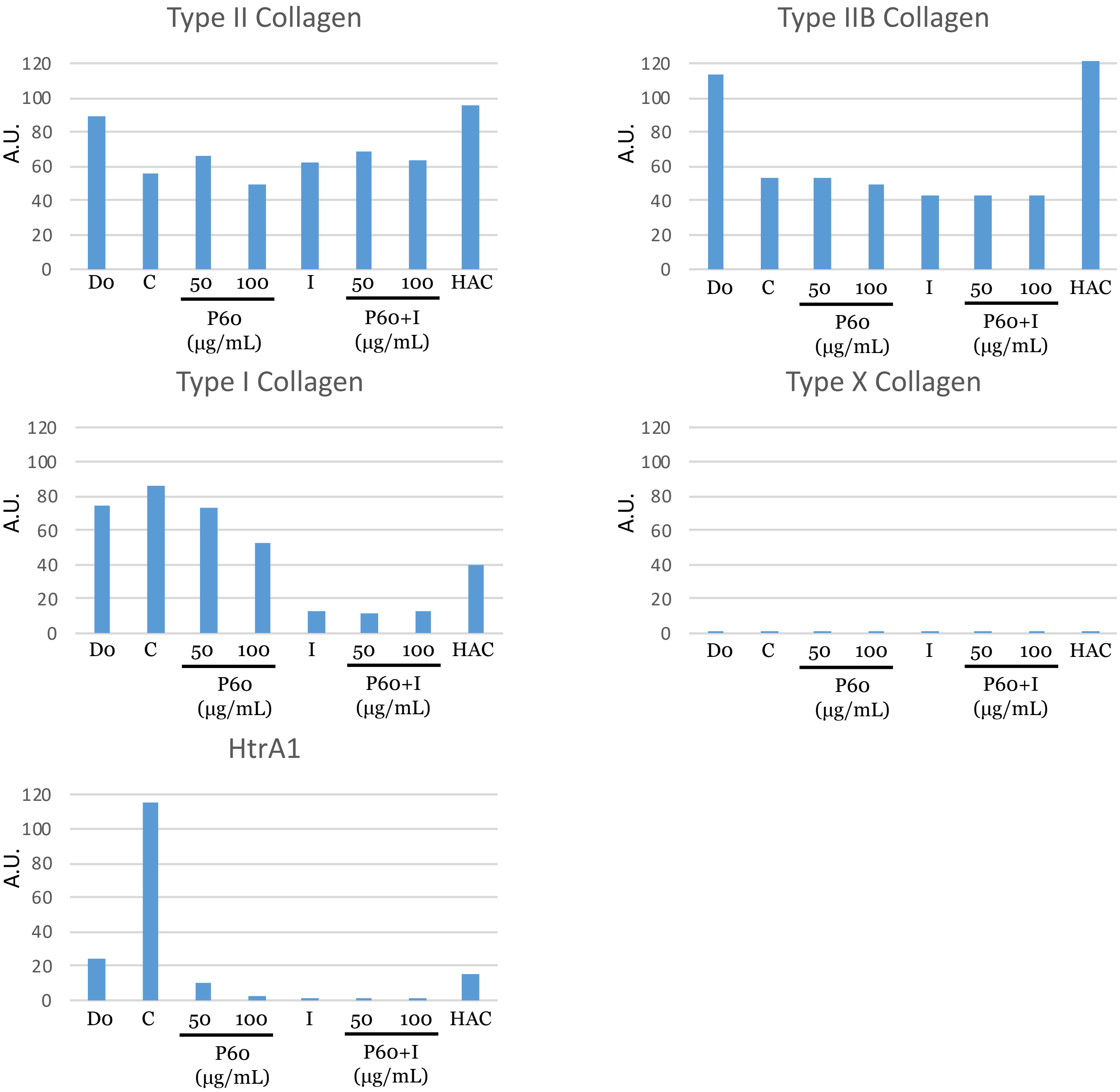

Figure S17C: Quantification of type II, IIB, I and X collagens and of Htra1 western-blots presented in Figure 10C. Protein expression was measured by quantifying the density of immunoblots bands calculated relative to GAPDH using image analysis software ImageJ®. A.U.: Arbitrary Unit.

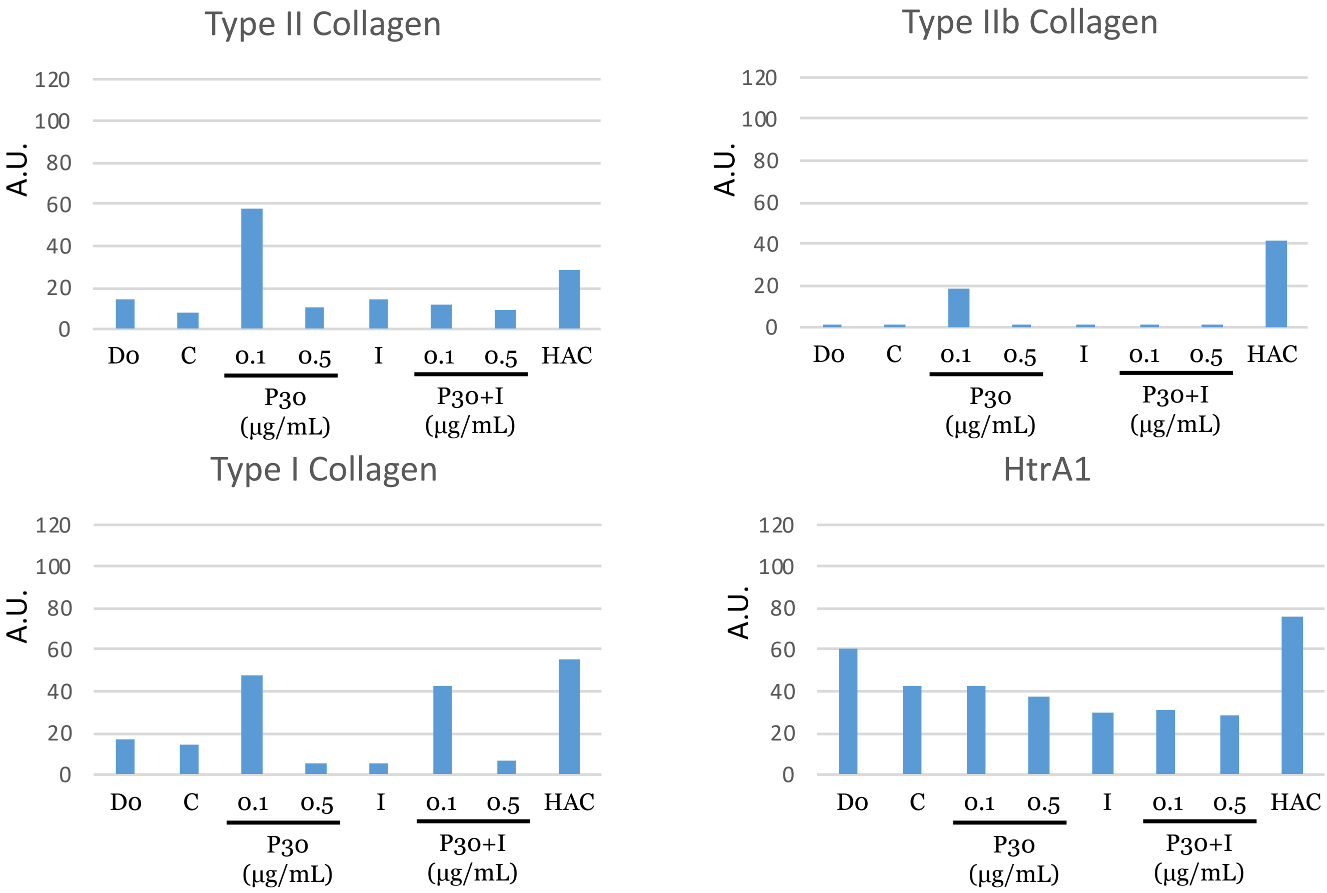

Figure S18A: Quantification of type II, IIB and I collagens and of Htra1 immunoblots presented in Figure S8A. Protein expression was measured by quantifying the density of immunoblots bands calculated relative to GAPDH using image analysis software ImageJ®. A.U.: Arbitrary Unit.

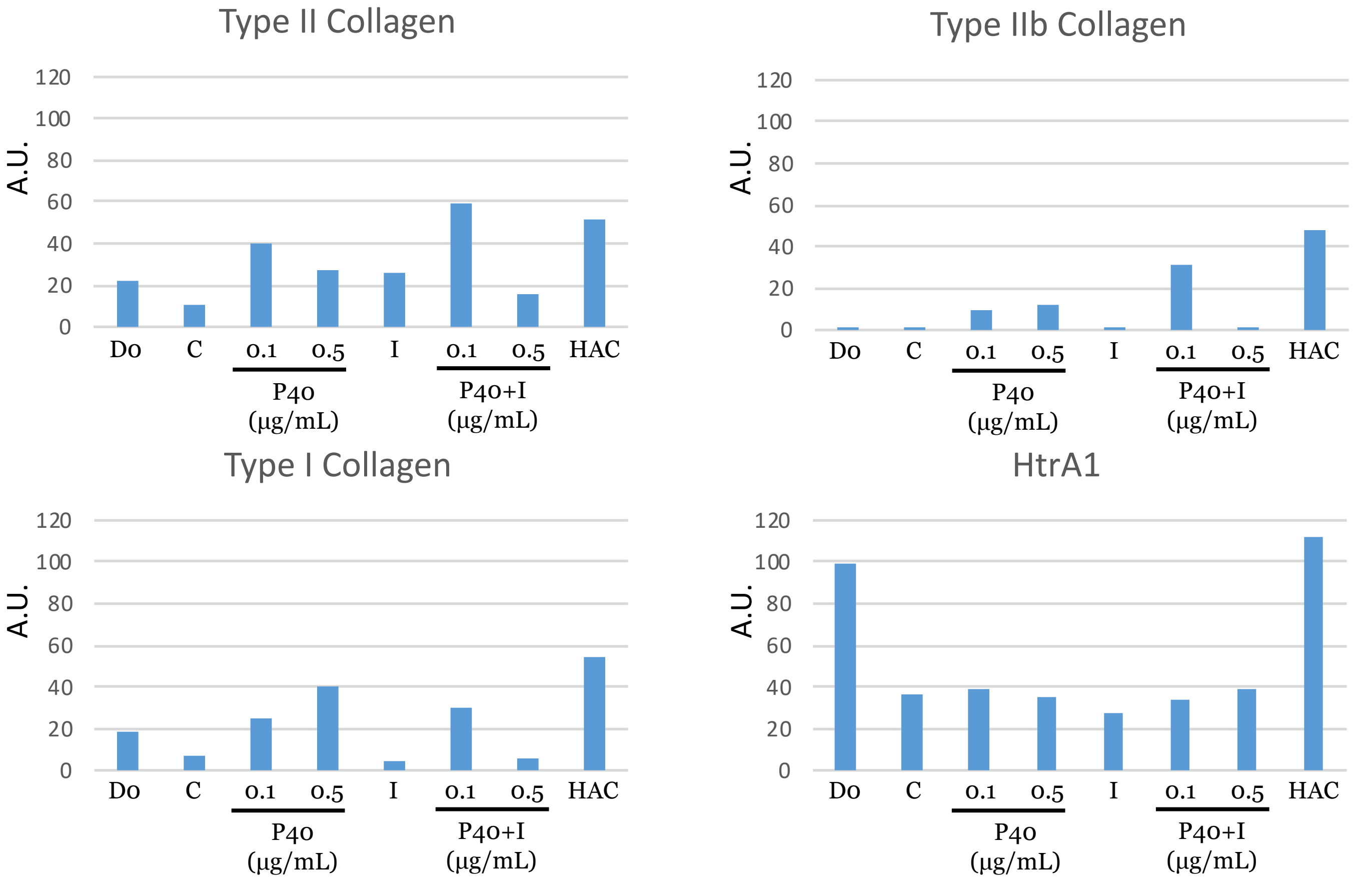

Figure S18B: Quantification of type II, IIB and I collagens and of Htra1 immunoblots presented in Figure S8B. The relative densitometry values (calculated relative to GAPDH) were determined using ImageJ®software. A.U.: Arbitrary Unit.

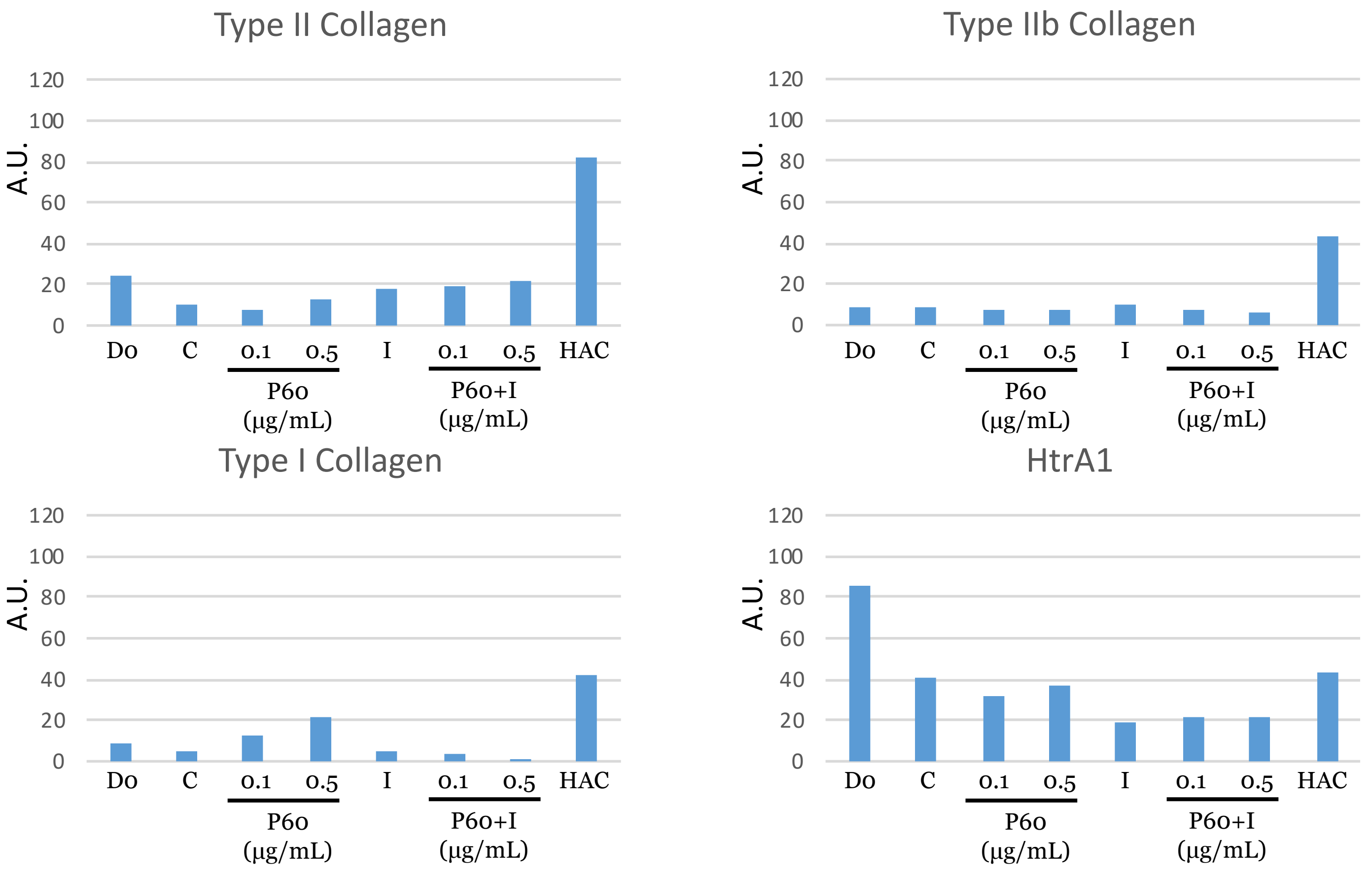

Figure S18C: Quantification of type II, IIB and I collagens and of Htra1 immunoblots presented in Figure S8C. The relative densitometry values (calculated relative to GAPDH) were determined using ImageJ®software. A.U.: Arbitrary Unit.

Gene	Sequence Forward	Sequence Reverse
ACAN	TCGAGGACAGCGAGGCC	TCGAGGGTGTAGCGTGTAGAGA
ß-ACTIN	CCGCGAGCACAGAGCCTCGCCTT	GGCCTTGCACATGCCGGAGCCGT
ADAMTS5	CCTCTCCCATGACGATTCCA	TGCTTTCGTGGTAGGTCCAG
BGLAP	GCAGCGAGGTAGTGAAGAGAC	AGCAGAGCGACACCCTA
COL1A1	TGAGCCAGCAGATCGAGA	ACCAGTCTCCATGTTGCAGA
COL1A2	TGTGGATACGCGGACTTTGT	CAGCAAAGTTCCCACCGAGA
COL2A1	GGCAATAGCAGGTTCACGTACA	CGATAACAGTCTTGCCCCACTT
COX-2	CCCTTCTGCCTGACACCTTT	ACTTTCTGTACTGCGGGTGG
GAPDH	CCTGCACCACCAACTGCTTA	GGCCATCCACAGTCTTCTGGG
HTRA1	GACTACATCCAGACCGACGC	GCTTTTCCTTTGGCCTGTCG
INOS	ACATTCTGCTTCTGGAAACTA	CGCTACAACATCCTGGAG
MMP1	GGCCCACAAACCCCAAAAG	ATCTCTGTCGGCAAATTCGTAAGC
MMP13	CCCCAACCCTAAACATCCAAAAAC	TTAAAAACAGCTCCGCATCAACCT
MMP14	GCCGGGGCATCCAGCAACTTTA	TCCTCACCCGCCAGAACCAG
MMP9	TGCCCGGACCAAGGATACAGTTT	AGGCCGTGGCTCAGGTTCAGG
P21	CATGTGGACCTGTCACTGTCTTGTA	GAAGATCAGCCGGCGTTTG
P53	CCCCAGCCAAAGAAGAAAC	AACATCTCGAAGCGCTCAC
P65	GCACAGATACCACCAAGACC	TCAGCCTCATAGAAGCCATC
PCNA	GGCCGAAGATAACGCGGATAC	GGCATATACGTGCAAATTCACCA
PPIA	CGGATTTGATCATTTGGTG	CAGGGAATACGTAACCAG
PRG4	GAACGTGCTATAGGACCTTC	CAGACTTTGGATAAGGTCTGCC
RUNX2	AGCCTTACCAAACAACACACCAG	CCATATGTCCTCTCAGCTCAGC
SOX9	GTACCCGCACTTGCACAAC	TCGCTCTCGTTCAGAAGTCTC
SPP1	TGGAAAGCGAGGAGTTGAATGG	GCTCATTGCTCTCATCATTGGC

Table S1: Oligonucleotides used in RT-qPCR experiments.

Antibody	supplier	Dilution
Type IIB collagen	Covalab, Villeurbanne, France	1/1500
Type II collagen	Novotec, Bron, France	1/1500
Type I collagen	Novotec, Bron, France	1/1500
Type X collagen	Sigma-Aldrich,St. Louis, MO, USA	1/1000
Htra1	ABGENT, San Diego, CA, USA	1/3000
GAPDH	Santa Cruz Biotechnology, Dallas, TX, USA	1/5000

Table S2: Antibodies used in the western-blots.
